# NOD-like receptor NLRC5 promotes neuroinflammation and inhibits neuronal survival in Parkinson’s disease models

**DOI:** 10.1186/s12974-023-02755-4

**Published:** 2023-04-18

**Authors:** Zhaolin Liu, Chenye Shen, Heng Li, Jiabin Tong, Yufei Wu, Yuanyuan Ma, Jinghui Wang, Zishan Wang, Qing Li, Xiaoshuang Zhang, Hongtian Dong, Yufang Yang, Mei Yu, Jian Wang, Renyuan Zhou, Jian Fei, Fang Huang

**Affiliations:** 1 Department of Translational Neuroscience, Jing’an District Centre Hospital of Shanghai; State Key Laboratory of Medical Neurobiology and MOE Frontiers Center for Brain Science, Institutes of Brain Science, Fudan University, 138 Yixueyuan Road, Shanghai, 200032 China; 2 Department of Neurology, Huashan Hospital, Fudan University, 12 Wulumuqi Zhong Road, Shanghai, 200040 China; 3 School of Life Science and Technology, Tongji University, 1239 Siping Road, Shanghai, 200092 China; 4 Shanghai Engineering Research Center for Model Organisms, Shanghai Model Organisms Center, INC., Shanghai, 201203 China

**Keywords:** Parkinson’s disease, NLRC5, Neuroinflammation, Microglia, Neuronal survival

## Abstract

Parkinson’s disease (PD) is mainly characterized by the progressive degeneration of dopaminergic neurons in the substantia nigra pars compacta (SNpc) and neuroinflammation mediated by overactivated microglia and astrocytes. NLRC5 (nucleotide-binding oligomerization domain-like receptor family caspase recruitment domain containing 5) has been reported to participate in various immune disorders, but its role in neurodegenerative diseases remains unclear. In the current study, we found that the expression of NLRC5 was increased in the nigrostriatal axis of mice with 1-methyl-4-phenyl-1,2,3,6-tetrahydropyridine hydrochloride (MPTP)-induced PD, as well as in primary astrocytes, microglia and neurons exposed to different neurotoxic stimuli. In an acute MPTP-induced PD model, NLRC5 deficiency significantly reduced dopaminergic system degeneration and ameliorated motor deficits and striatal inflammation. Furthermore, we found that NLRC5 deficiency decreased the expression of the proinflammatory genes *IL-1β*, *IL-6*, *TNF-α* and *COX2* in primary microglia and primary astrocytes treated with neuroinflammatory stimuli and reduced the inflammatory response in mixed glial cells in response to LPS treatment. Moreover, NLRC5 deficiency suppressed activation of the NF-κB and MAPK signaling pathways and enhanced the activation of AKT–GSK-3β and AMPK signaling in mixed glial cells. Furthermore, NLRC5 deficiency increased the survival of primary neurons treated with MPP^+^ or conditioned medium from LPS-stimulated mixed glial cells and promoted activation of the NF-κB and AKT signaling pathways. Moreover, the mRNA expression of *NLRC5* was decreased in the blood of PD patients compared to healthy subjects. Therefore, we suggest that NLRC5 promotes neuroinflammation and dopaminergic degeneration in PD and may serve as a marker of glial activation.

**Supplementary Information:**

The online version contains supplementary material available at 10.1186/s12974-023-02755-4.

## Introduction

Parkinson’s disease (PD), which is the second most common neurodegenerative disorder, is characterized by dopaminergic neuron lesions in the substantia nigra pars compacta (SNpc), reductions in dopamine (DA) levels in the striatum and motor impairments [1], and its prevalence is expected to increase worldwide [2, 3]. In addition to Lewy bodies formed by misfolded α-synuclein proteins [4], accumulating evidence suggests that neuroinflammation, which is one of the core pathological hallmarks of PD [3, 5], is mediated by microglia [6, 7] and astrocytes [8, 9] in the central nervous system (CNS). Microglia, which are the resident macrophages in the CNS, engage in bidirectional communication with astrocytes [10], releasing proinflammatory cytokines such as interleukin-1β (IL-1β) and nitric oxide (NO) and reactive oxygen species (ROS) under pathological conditions, including in experimental PD models induced by 1-methyl-4-phenyl-1,2,3,6-tetrahydropyridine (MPTP)-, LPS-, and α-synuclein [11–15]. Most studies have concluded that chronic neuroinflammation caused by activated microglia and astrocytes is harmful to dopaminergic neurons in the brains of individuals with PD [12].

NLR (nucleotide-binding domain, leucine-rich repeat-containing) proteins are widely involved in inflammatory processes, including inflammasome assembly, the innate immune response, and transcriptional activation in human diseases [16, 17]. NLRC5 is a member of the NLR family that contains an N-terminal caspase activation and recruitment domain (CARD), a conserved central NACHT (named for the NAIP, CIITA, HET-E, and TP-1 proteins) domain and a C-terminal leucine-rich repeat (LRR) domain [18]. NLRC5 has been identified as a regulator of NF-κB and a key transcriptional activator of the MHCI gene in immune cells or immune-related tissues, revealing its potential role in the regulation of inflammation and innate immunity [19–22]. The expression of NLRC5 can be induced by immune-related stimuli, such as lipopolysaccharide (LPS), poly(I:C), interferon (IFN), or other pathogen-associated molecular patterns (PAMPs), including viral infection. However, the functions of NLRC5 in different inflammatory conditions and diseases are still unclear. For example, in RAW264.7 cells (a murine macrophage cell line) stimulated with LPS or lipoteichoic acid (LTA), knockdown of Nlrc5 by siRNA enhanced NF-κB activation [20, 23, 24]. In LX-2 human hepatic stellate cells, knockdown of *Nlrc5* increased NF-κB activation after TNF-α treatment [25], whereas in the human monocytic cell line THP-1, knockdown of *Nlrc5* eliminated IL-1β processing in response to bacterial infection and downregulated the poly(I:C)-mediated type I interferon pathway [26, 27]; the same effect was observed in virus-infected human foreskin fibroblasts [28]. Recent studies on renal ischemia/reperfusion (I/R) models showed that *Nlrc5* deficiency reduced inflammatory responses in the kidneys, as indicated by reduced expression of the proinflammatory molecules IL-1β, IL-6, and TNF-α and reduced levels of neutrophil, macrophage, dendritic cell and CD4^+^ T cell infiltration [29]. A diabetic nephropathy model showed that *Nlrc5* deficiency reduced the inflammatory response, suppressed the NF-κB pathway and reduced macrophage infiltration in the diabetic kidney [30]. Therefore, the role of NLRC5 in inflammatory responses appears to be highly tissue/cell type- and stimulation dependent.

NF-κB activation regulates inflammation and cell survival/death in neurological disorders and many other diseases [31, 32]. Recent work has revealed that inhibiting NF-κB signaling and the activation of NRF2 signaling in glial cells support dopaminergic neuronal survival [33]. In addition, NLRC5 has been reported to modulate hippocampal neuronal survival through an NRF2-related pathway [34]. Knockdown of *Nlrc5* promotes the AKT signaling pathway [35] and contributes to cell survival by downregulating apoptosis-related molecules [36]. However, the role of NLRC5 in dopaminergic neuronal death in PD remains unclear.

In this study, altered *NLRC5* expression was detected in the peripheral blood of PD patients, and we demonstrated that NLRC5 could positively regulate neuroinflammation and suppress neuronal survival in an MPTP-induced PD mouse model and MPP^+^/LPS-induced cellular PD models. The roles of NLRC5 in the regulation of the NF-κB and AKT signaling pathways were further revealed.

## Materials and methods

### Animals and treatments

All experimental protocols were approved by the Institutional Animal Care and Use Committee of Fudan University, Shanghai Medical College. *Nlrc5*^−/−^ (knockout, KO) mice and littermate control (wild-type, WT) mice weighing between 25 and 30 g were obtained from Shanghai Model Organisms Center, Inc. (Shanghai, China) and were generated with the C57BL/6 strain as previously described [37]. KO and WT mice were maintained under a 12-h light–dark cycle in temperature-controlled rooms (18–22 °C), with libitum access to food and water.

For the acute 1-methyl-4-phenyl-1,2,3,6-tetrahydropyridine (MPTP)-induced mouse model, male WT and KO mice were divided into MPTP-treated groups and normal saline (NS)-treated groups and were intraperitoneally injected with MPTP–HCl (15 mg/kg, Sigma‒Aldrich, USA) or equal volumes of normal saline 4 times at 2-h intervals in a single day. Each group contained 4–11 mice, some of the striatal samples were processed for high-performance liquid chromatography (HPLC) analysis, and the other half of the brains were randomly used for immunoblotting, qPCR analysis, immunohistochemistry or immunofluorescence staining.

### Protein extraction and immunoblot analysis

Animal tissues or cell samples were homogenized with 1 × RIPA buffer (Thermo Scientific, USA) containing protease and phosphatase inhibitor cocktails (Thermo Scientific, USA), and the extracted protein concentration was determined using a BCA kit (Thermo Scientific, USA). After the samples were boiled in protein loading buffer, 30 μg of protein per sample was loaded onto sodium dodecyl sulfate‒polyacrylamide gel electrophoresis (SDS‒PAGE) gels, transferred to polyvinylidene difluoride (PVDF) membranes with pore size of 0.45 μm, and blocked with 5% nonfat dry milk in 1 × Tris-buffered saline containing 0.1% Tween-20 (TBST) for 1 h. Then, the membranes were incubated overnight at 4 °C with the following primary antibodies: rabbit anti-NLRC5 (Abcam, 1:1000), rabbit anti-tyrosine hydroxylase (TH, Abcam, 1:1000), mouse anti-glial fibrillary acidic protein (GFAP, Proteintech Group, 1:1000), rabbit anti-IL-1β (Abcam, 1:1000), rabbit anti-COX-2 (Abcam, 1:1000), rabbit anti-phospho-NF-κB p65 (Ser536, p-P65, Cell Signaling Technology, 1:1000), rabbit anti-phospho-IKKα/β (Ser176/180, p-IKKα/β, Cell Signaling Technology, 1:1000), rabbit anti-phospho-Akt (Thr308, Cell Signaling Technology, 1:1000), rabbit anti-phospho-GSK3 beta (Ser9, p-GSK3β, Affinity, 1:1000), rabbit anti-phospho-GSK3 beta (Ser9, p-GSK3β, Affinity, 1:1000), rabbit anti-phospho-AMPK alpha (Thr172, Affinity, 1:1000), rabbit anti-phospho-ERK1/2 (Thr202/Tyr204, p-ERK1/2, Affinity, 1:1000), rabbit anti-phospho-JNK (Thr183 + Tyr185, p-JNK, Affinity, 1:1000), rabbit anti-phospho-p38 MAPK (Thr180/Tyr182, p–p38, Affinity, 1:1000), and anti-β-actin (Santa Cruz, 1:2000). Then, the blots were washed and incubated with appropriate secondary antibodies (LI-COR, 1:10,000) at room temperature for 1 h. After the blots were washed with TBST, the protein signals were detected using an infrared imaging system (LI-COR) and quantified by densitometric analysis using Quantity One software (Bio-Rad, USA).

### Immunohistochemistry and immunofluorescence analysis

The brain sections were cut to a thickness of 30 μm using a frozen microtome (Leica, Germany). For immunohistochemistry, the sections were treated with phosphate-buffered saline (PBS) containing 0.6% H_2_O_2_ to eliminate endogenous peroxidase activity, permeabilized with 0.5% Triton X-100 in PBS for 1 h and blocked in PBS with 10% normal goat serum at room temperature (RT) for 1 h. The sections were then incubated with primary antibodies (mouse anti-TH, Sigma, 1:1000; rabbit anti-Iba1, Abcam, 1:1000; mouse anti-GFAP, Millipore, 1:1000) in PBS containing 1% goat serum at 4 °C overnight. After being washed with PBS (3 times, 10 min each), the sections were incubated with biotin-conjugated secondary antibodies (1:200) for 1 h at 37 ℃, followed by AB peroxidase (1:200 for each, Vector Laboratories, USA) treatment for 45 min at RT. Signals were detected using a 3,3′-diaminobenzidine kit (DAB, Vector Laboratories, USA). Images of the stained sections were obtained by bright-field microscopy (OLYMPUS, Japan), and the optical density (OD) of striatal TH-positive fibers was quantitatively determined using Image-Pro Plus 6.0 software (Media Cybernetics, USA).

For immunofluorescence staining, the sections were permeabilized and blocked without H_2_O_2_ treatment and then incubated with primary antibodies (mouse anti-NLRC5, Santa Cruz, 1:500; goat anti-CD16, R&D System, 1:500; the same catalog numbers and dilutions as anti-TH, anti-Iba1 and anti-GFAP) at 4 °C overnight. After being washed with PBS, the sections were incubated with secondary antibodies (Alexa Fluor 594-conjugated goat anti-mouse IgG and Alexa Fluor 488-conjugated goat anti-rabbit IgG, Thermo Fisher, 1:1000) at RT for 1 h without light. Images of the stained sections were obtained by confocal microscopy (Nikon, Japan).

### RNA extraction and quantitative real-time PCR

Total RNA was extracted from cells, tissues or blood samples using TRIzol reagent (Tiangen, China) according to the manufacturer’s instructions. The concentration of each RNA sample was determined by measuring the absorbances at 260 and 280 nm by a spectrophotometer (Biotek, USA). No more than 2000 ng of RNA was reverse-transcribed into complementary DNA (cDNA) using an RT Super-Mix kit (Tiangen, China). Real-time PCR (RT‒qPCR) was performed to quantify target gene levels with a quantitative thermal cycler (Eppendorf, Germany). Gene expression was normalized to that of β-actin, and the expression level was calculated using the 2^−ΔΔCt^ method. The primers used for RT‒qPCR are listed in Additional file 1: Table S1.

### High performance liquid chromatography (HPLC)

The dissected striatum tissues were homogenized with a high-speed homogenizer (MP Biomedicals, USA) at a speed of 5 m/s in 0.4 M HClO_4_ for 30 s and then centrifuged at 12,000*g* and 4 °C. The supernatants were collected to determine the concentrations of DA and its metabolites homovanillic acid (HVA) and 3,4-dihydroxyphenylacetic acid (DOPAC), as well as serotonin (5-HT), using a chromatograph (ESA, USA) with a 5014B electrochemical detector.

### Cell culture and treatments

Primary cultures of murine astrocytes and microglia were generated as described previously [13]. The brains of newborn WT and KO mice were dissected under sterile conditions in Hank’s salt (HBSS), and the meninges and blood vessels were removed. Brain samples were mechanically dissociated into single cell suspensions with precooled flowing HBSS, plated on 75 cm^2^ poly-d-lysine-coated flasks (Corning, USA), and incubated in Dulbecco’s modified Eagle’s medium (DMEM) containing 10% fetal bovine serum (FBS) and 50 U/mL penicillin/streptomycin at 37 °C and 5% CO_2_. The medium was changed every 48 h. On the 14th day, loosely adherent microglial cells were shaken off the confluent astrocytes at 220 rpm for 4 h to harvest enriched microglia and astrocytes, which were replated in 24-well plates (2.5 × 10^5^ cells/well, 3 wells per group) and 6-well plates (1 × 10^6^ cells/well, 6 wells per group), respectively. For mixed glial cells, the cells in the flasks were digested and replated in 6-well plates (1 × 10^6^ cells/well, 6 wells per group).

Primary neurons were isolated from mouse embryos 13.5–14 days after gestation as previously described [38]. For PI staining, neuronal cells were plated on sterile glass pieces (14 mm in diameter, 9 × 10^4^ cells/piece, 6 wells per group) in poly-D-lysine (PDL, 50 μg/mL, Sigma)-precoated 24-well plates for 2 h at 37 ℃. For RNA and protein extraction, neuronal cells were plated in 6-well plates (PDL-precoated, 1 × 10^6^ cells/well, 6 wells per group). Cells were cultured in DMEM containing 10% FBS for the first 2 h to allow for attachment, and then the medium was replaced with neurobasal medium (Thermo Scientific, USA) containing 2% B27 (Thermo Scientific, USA). Neuronal cells were cultured at 37 °C and 5% CO_2_ for 6 days before treatment, during which the neurobasal medium was half changed every 48 h.

SH-SY5Y cells and BV-2 cells were maintained in DMEM supplemented with 10% FBS and antibiotics under the same conditions and were replated in 6-well plates (1 × 10^6^ cells/well) 24 h before being treated.

BV-2 cells were treated with PBS or 100 ng/mL LPS for 12 h, and the supernatant was collected and centrifuged at 5000*g* to eliminate cell debris and was used as conditioned medium (B-CM) and LPS-stimulated conditioned medium (B-LCM).

Mixed glial cells were treated with PBS or 250 ng/mL LPS for 24 h, and the supernatant was collected and centrifuged at 5000×*g* to eliminate cell debris and was used as conditioned medium (CM) and LPS-stimulated conditioned medium (LCM).

After returning to the resting state (72–96 h after replating), microglia in 24-well plates were challenged with PBS or 100 ng/mL LPS. Astrocytes in 6-well plates were treated with 1 mM MPP^+^ or B-LCM, and control groups were treated with equal volumes of PBS or B-CM. Mixed glial cells were exposed to PBS or 250 ng/mL LPS. The samples were harvested 6 h later for total RNA extraction and 24 h later for protein analysis. The supernatant of mixed glial cells was also collected for NO and cytokine analysis. Primary neurons were treated with PBS, 20 μM MPP^+^ or CM and LCM for 24 h, and then PI staining and RNA and protein extraction were carried out.

### Stereological counting of TH^+^ cells

The total number of TH^+^ neurons in the SNpc was counted using a Stereo Investigator system (Micro Brightfield, USA) with bright-field microscopy (Olympus, Japan), as described previously[13, 39]. A total of six sections from the bregma − 2.80 to − 3.65 mm were collected and counted in real time under a 40× objective. Stereological counting was performed in a double-blind fashion.

### Cytokine and NO assays

The supernatants from mixed glial cells were collected, and cellular debris was eliminated through centrifugation at 5000×*g* for 5 min, after which the samples were aliquoted and stored at − 80 ℃. IL-1β levels in the supernatants were measured by murine ELISA kits (ABclonal Biotechnology, China) according to the manufacturer’s instructions. Briefly, 100 μL of standard and samples were added into designated wells and incubated at 37 °C for 2 h. After discarding the liquid, the wells were washed with wash buffer at least 3 times. Then, 100 μL of biotin-conjugated antibody solution was added to each well and incubated at 37 °C for 1 h. After being washed, each well was incubated with 100 μL of streptavidin–HRP solution at 37 °C for 30 min. Next, the wells were washed as described above and incubated with 100 μL of TMB substrate at RT for 20 min. Finally, 50 μL of stop solution was added, and the optical density was measured at 450 nm on a microplate reader (Epoch, BioTek, USA) within 5 min.

The levels of NO in the cultures were determined by measuring nitrite concentrations in supernatants using Griess reagent (Beyotime, China) according to the manufacturer’s instructions, and absorbance was measured at 540 nm on a microplate reader (Epoch, BioTek, USA). The nitrite concentration was calculated with reference to the standard curve generated with NaNO_2_.

### Behavioral tests

#### Open field test (OFT)

Three days after MPTP administration, locomotor behavior was assessed in the open field test with an automatic-recording open-field working station (MED Associates, USA). The total movement distance and average movement speed were recorded and analyzed within 10 min.

#### Rearing test

The rearing test was carried out 7 days after MPTP administration to assess the spasticity of mice. Small transparent cylinders (12 cm in diameter, 20 cm in height) were used. Each mouse was placed in a cylinder, and the number of rearings (forepaw touches to the cylinder) was manually scored within 3 min by an experimenter who was blinded to the genotype and treatment of the mice.

#### Pole test

A vertical pole (10 mm in diameter, 80 cm in height) with a rough surface was used. The mice were habituated to the task 1 day before testing, and those that could not turn around were corrected or rejected. Seven days after MPTP administration, the mice were placed head-up near the top of the pole, and the time to turn around and time to descend (climb down) were measured. The test was conducted in triplicate with a 30 min interval, and the average values were used for each animal.

### Patients and clinical assessments

Nineteen patients with PD and eighteen healthy subjects were recruited from the Department of Neurology, Huashan Hospital, Fudan University. The PD subjects were clinically examined and diagnosed by two senior investigators of movement disorders according to the UK Brain Bank criteria. All participants provided written informed consent in accordance with the Declaration of Helsinki. The study was approved by the Human Studies Institutional Review Board, Huashan Hospital, Fudan University. All methods were performed in accordance with the relevant guidelines and regulations. The demographic and clinical data of the patients and controls are summarized in Additional file 1: Table S2. Receiver operating characteristic (ROC) curve analysis was used to analyze the diagnostic performance of *NLRC5* and *CIITA* mRNA levels and determine the cutoff that maximized the sum of the specificity and sensitivity.

### Statistical analysis

**Fig. 1:**
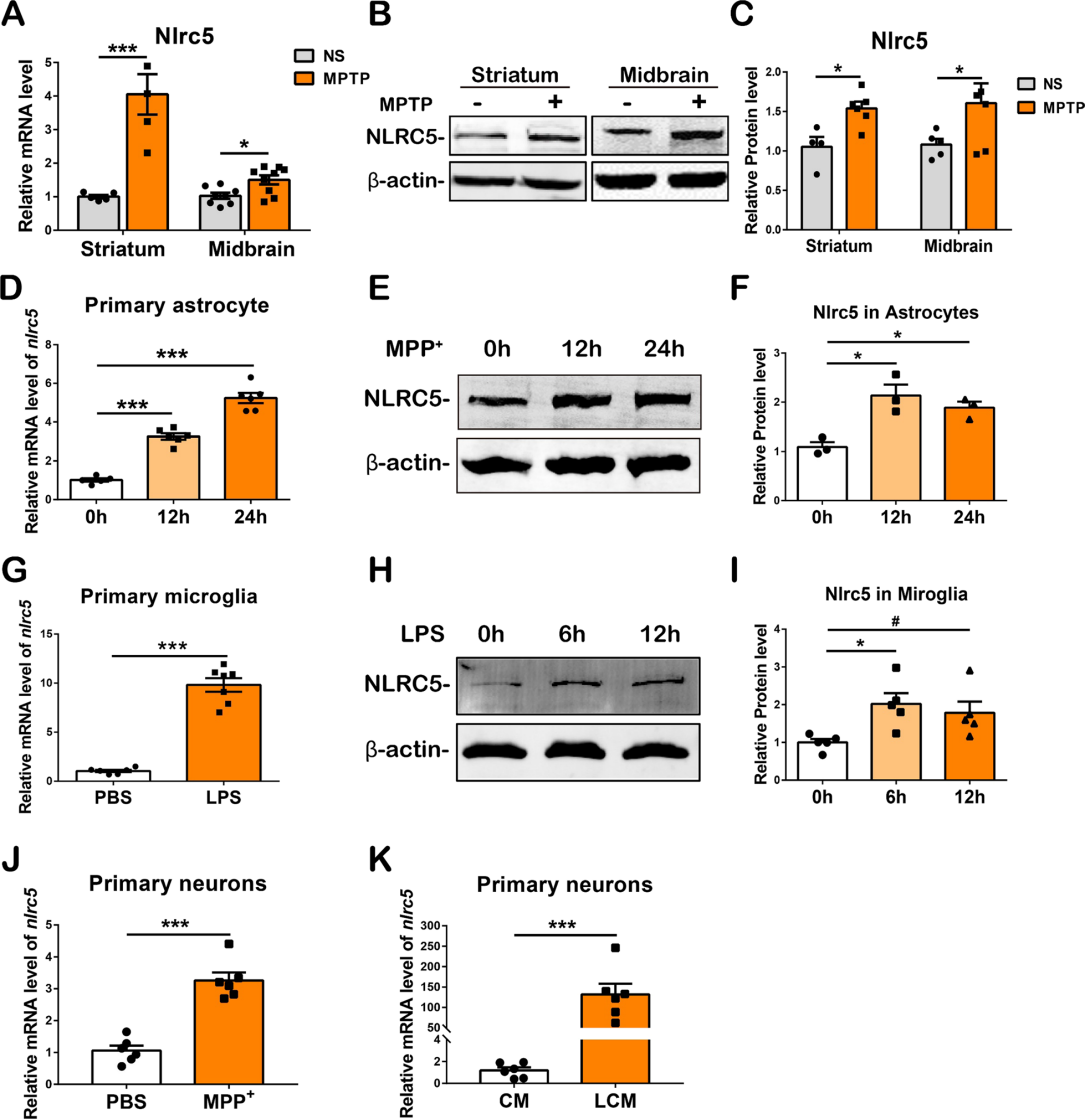
Expression of NLRC5 responses to various stimuli in vivo and in vitro. **A** Transcriptions of *Nlrc5* in the striatum and the midbrain at 3 days after NS or MPTP administration. **B**, **C** Protein levels of NLRC5 in the striatum and the midbrain detected by Western Blot at 7 days after NS or MPTP administration. **D** Transcriptions of *Nlrc5* in primary astrocytes treated with 1 mM MPP^+^ for 0 h, 12 h or 24 h. **E**, **F** Protein levels of NLRC5 in primary astrocytes treated with 1 mM MPP^+^ for 0 h, 12 h or 24 h. **G** Transcriptions of *Nlrc5* in primary microglia treated with 100 ng/mL LPS for 0 h or 6 h. **H**, **I** Protein levels of NLRC5 in primary microglia treated with 100 ng/mL LPS for 0 h, 6 h or 12 h. **J** Transcriptions of *Nlrc5* in primary neurons treated with PBS or 20 μM MPP^+^ for 24 h. **K** Transcriptions of Nlrc5 in primary neurons treated with conditioned medium (CM) or LPS-treated conditioned medium (LCM) from mixed glial cells for 24 h. Statistical analyses were performed with Student’s t test (**A**, **C**, **G**, **J**, **K**), or one-way ANOVA followed by Holm–Sidak’s multiple comparisons test (**D**, **F**, **I**). *n* = 3–9. *p < 0.05, **p < 0.01, and ***p < 0.001

The data were analyzed using Prism 7.0 software (GraphPad Software, USA). All values are expressed as the means ± SEMs and were assessed for normal distribution by the Shapiro‒Wilk test. Unpaired two-tailed Student's *t* test was used for two-group comparisons (Figs. 1A, C, G, I–K, 9, Additional file 1: Fig. S1 B and D), one-way ANOVA followed by Holm‒Sidak’s multiple comparisons test was used to analyze the effects of treatments at different timepoints (Fig. 1D, F and I, and two-way ANOVA followed by Holm‒Sidak’s multiple comparisons test was used for comparisons among genotypes and treatments. Statistically significant differences were defined as *p* < 0.05.

## Results

### The expression of NLRC5 is regulated by MPTP, MPP^+^ and LPS stimulation in vivo and in vitro

The expression of NLRC5 in the nigrostriatal system and primary neural cells was investigated. The transcription of *Nlrc5* was significantly elevated in the striatum and the ventral midbrain 3 days after MPTP administration, as detected by RT‒qPCR (Fig. 1A). Immunoblot analysis showed that NLRC5 protein expression was increased in the striatum and the ventral midbrain after MPTP administration (Fig. 1B, C). In addition to that in the nigrostriatal system, the expression of NLRC5 was also observed in the hippocampus and the cortex (Additional file 1: Fig. S1A, B). Immunostaining showed that NLRC5^+^ signals were present in astrocytes, microglia and dopaminergic neurons (Additional file 1: Fig. S1C). Enriched primary cultures of these three cell types were obtained for in vitro research. MPP^+^, a neurotoxic metabolite of MPTP, was used to stimulate primary astrocytes, mimicking the reactivation of astrocytes in vivo. The mRNA and protein expression levels of NLRC5 in primary astrocytes were dramatically increased at 12 h and 24 h after MPP^+^ stimulation, as shown by RT‒qPCR and immunoblotting, respectively (Fig. 1D–F). Lipopolysaccharide (LPS) is commonly used to activate microglial cells. The transcription of *Nlrc5* was upregulated in primary astrocytes 24 h after treatment with LPS-induced BV2 conditioned medium (B-LCM), but there was no significant difference (Additional file 1: Fig. S1D). Moreover, the transcription of *Nlrc5* in primary microglia was elevated 6 h after LPS treatment (Fig. 1G), and a twofold increase in NLRC5 protein was detected at 6 h and 12 h after LPS treatment (Fig. 1H, I). To imitate the effects of the neurotoxic milieu on neurons in MPTP-challenged mice, MPP^+^- and LPS-induced mixed glial cell medium (LCM) were used to stimulate primary neurons. The RT‒qPCR results revealed significant upregulation of *Nlrc5* transcription in neurons 24 h after the treatments (Fig. 1J, K). Notably, immunocytochemistry (ICC) demonstrated that NLRC5 was mainly distributed in the cytoplasm of SH-SY5Y cells and gradually translocated into the nucleus after MPP^+^ treatment, reaching a peak at 3–6 h (Additional file 1: Fig. S1E). These data indicate that NLRC5 is extensively expressed in the nigrostriatal axis and is highly induced by MPTP in vivo and neuroinflammatory stimuli in vitro.

### Nlrc5 deficiency reduces dopaminergic neuronal damage in the nigrostriatal axis of MPTP-treated mice

**Fig. 2:**
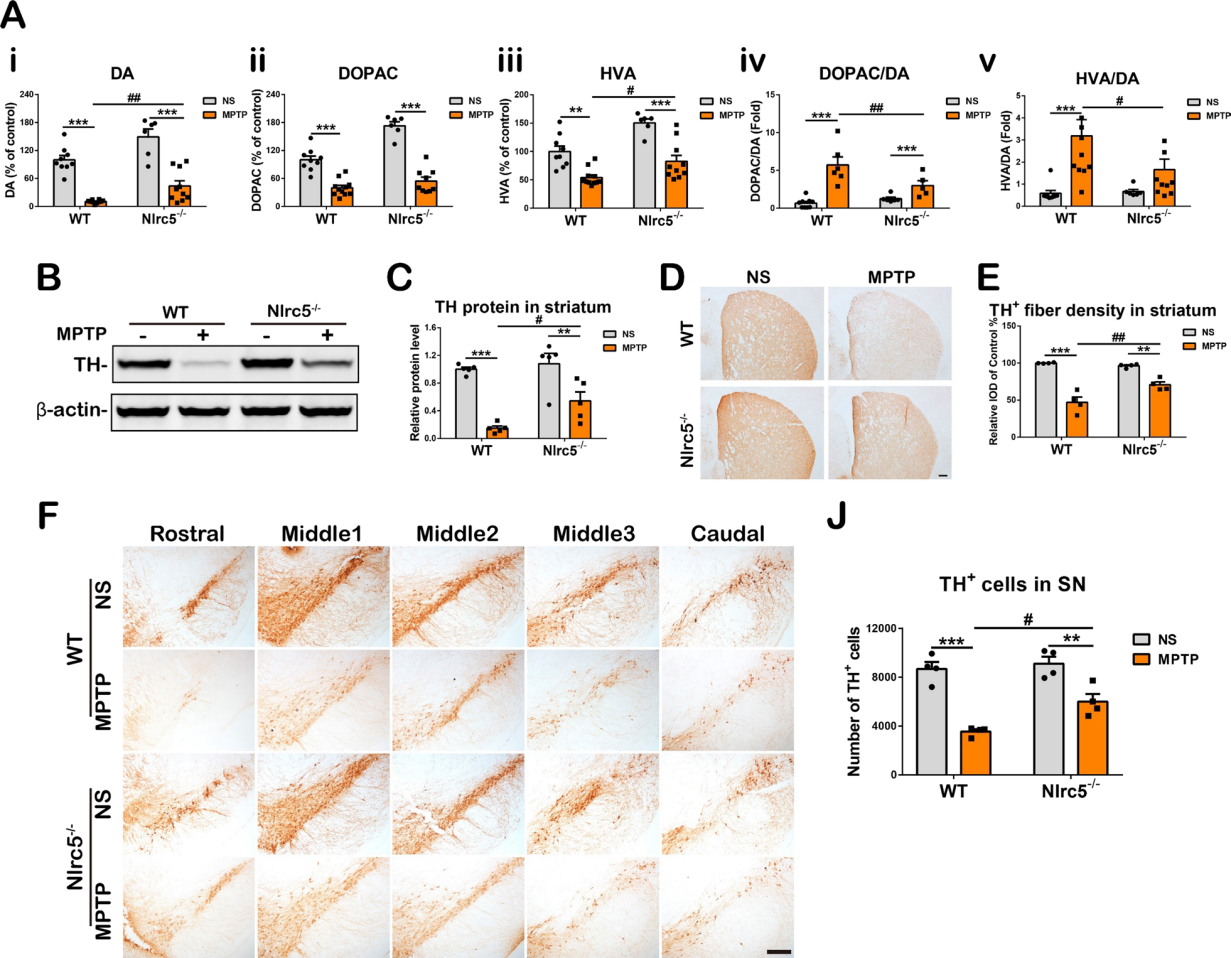
*Nlrc5* deficiency ameliorates MPTP-induced dopaminergic neuronal damage in the nigrostriatal axis 7 days after MPTP administration. **A** HPLC assays of DA (**i**), DOPAC (**ii**), and HVA **(iii)** presented by relative values, the ratio of DOPAC and HVA to DA were calculated (**iv**, **v**). n = 6–11. **B**, **C** Immunoblotting analysis of TH proteins in the striatum. *n* = 4–5. **D**, **E** Immunohistochemistry staining and optical density analysis of TH^+^ nerve fibers in the striatum. Scale bar, 200 μm. **F**, **J** Immunohistochemistry staining and stereological counting of TH^+^ neurons in the SNpc. Scale bar, 200 μm. *n* = 4. ^#^*p* < 0.05, ** or ^##^*p* < 0.01, and ***p < 0.001

To address the function of *Nlrc5* in PD, *Nlrc5*-knockout mice (*Nlrc5*^*−/−*^ or KO) were used for further study [37]. Nissl staining showed that the loss of *Nlrc5* caused no obvious structural alterations in the striatum, hippocampus, SN or cortex (Additional file 1: Fig. S2). MPTP is a neurotoxin that is commonly used in PD studies that selectively damages dopaminergic neurons and induces PD-like symptoms and neuroinflammation in mice [40]. In this study, an acute MPTP regimen (4 intraperitoneal injections of MPTP at 2 h intervals) was used as previously described [41]. Seven days after MPTP administration, HPLC was performed to evaluate the levels of the neurotransmitter dopamine in the striatum. The striatal levels of DA and its metabolites DOPAC and HVA [Fig. 2A(i–iii)] were sharply reduced in MPTP-treated mice; however, the reduction in DA was attenuated in MPTP-treated *Nlrc5*^*−/−*^ mice [Fig. 2A(i)]. The metabolic rate of DA was represented by the ratios of DOPAC and HVA to DA. Mice with *Nlrc5* deficiency exhibited a lower DA metabolic rate than WT mice after MPTP administration [Fig. 2A(iv and v)]. Moreover, MPTP administration resulted in a prominent decrease in striatal TH protein levels, and this decrease was mitigated in *Nlrc5*^*−/−*^ mice (Fig. 2B, C). Immunohistochemical staining and optical density analysis showed that *Nlrc5*^*−/−*^ mice exhibited attenuated depletion of TH^+^ nerve fibers in the striatum after MPTP administration (Fig. 2D and E). Immunohistochemical staining and stereological cell counting showed that the administration of MPTP significantly decreased the number of TH^+^ neurons in the SNpc of WT and KO mice, and *Nlrc5* deficiency ameliorated the decrease to 34% compared to 59% in WT mice (Fig. 2F, J). Thus, *Nlrc5* deficiency markedly decreased MPTP-induced dopaminergic system impairments in mice.

### *Nlrc5* deficiency ameliorates motor deficits in MPTP-treated mice

**Fig. 3:**
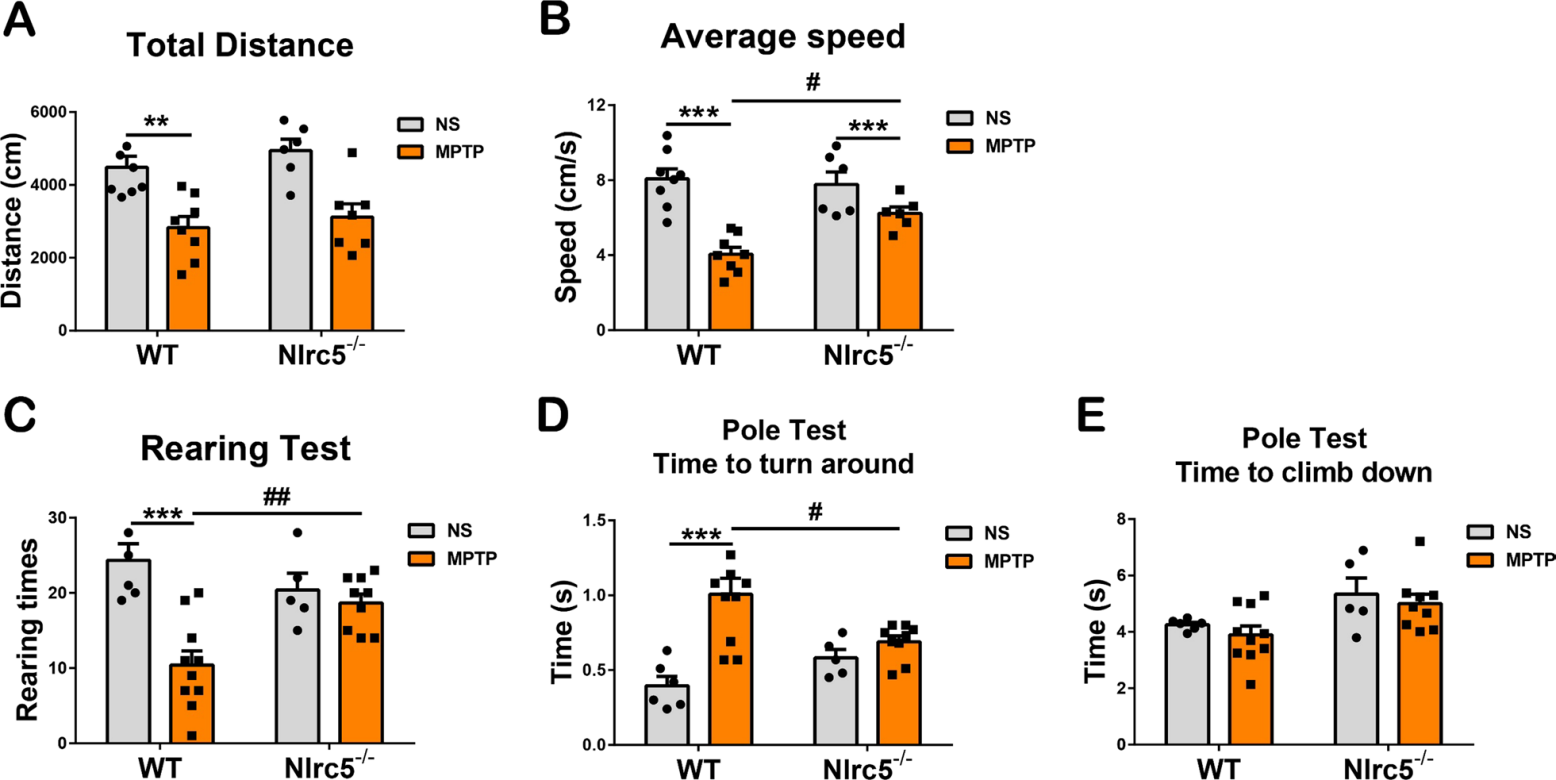
Behavioral assessments in WT and *Nlrc5*^*−/−*^ mice after MPTP administration. **A** Total moving distance in the Open field test. **B** Average moving speed in the OFT. **C** Rearing times in the Rearing test. **D** Time to turn around in the Pole test. **E** Time to climb down in the Pole test. All data were presented as the means ± SEM. *n* = 5–10. ^#^*p* < 0.05, ** or ^##^*p* < 0.01, and ***p < 0.001

To evaluate the effect of *Nlrc5* deficiency on MPTP-induced motor deficits, behavior tests, including the open field test (OFT), rearing test and pole test, were carried out. The OFT is a widely used paradigm for evaluating locomotor behavior that can be used to assess the motor function of PD mice [42]. Three days after MPTP administration, the OFT was performed and demonstrated that the total movement distance and the average speed were significantly reduced in WT mice but not in *Nlrc5*^*−/−*^ mice (Fig. 3A, B). Seven days after MPTP administration, the rearing test was performed to assess the rigidity degree of PD mice. Due to upper limb rigidity caused by MPTP, WT mice showed a twofold reduction in rearing times; however, the rearing times were not affected in MPTP-treated *Nlrc5*^−/−^ mice (Fig. 3C). In addition, the pole test was performed to investigate whether *Nlrc5* deficiency had an effect on motor initiation and coordination. MPTP treatment prolonged the time to turn around in WT mice but not in *Nlrc5*^−/−^ mice (Fig. 3D). MPTP administration had no effect on motor coordination, as the time to climb down was not different among the two genotypes (Fig. 3E). Therefore, *Nlrc5* deficiency alleviated MPTP-induced behavioral impairments in mice.

### *Nlrc5* deficiency reduces microglia and astrocyte activation in the nigrostriatal axis of MPTP-treated mice

**Fig. 4:**
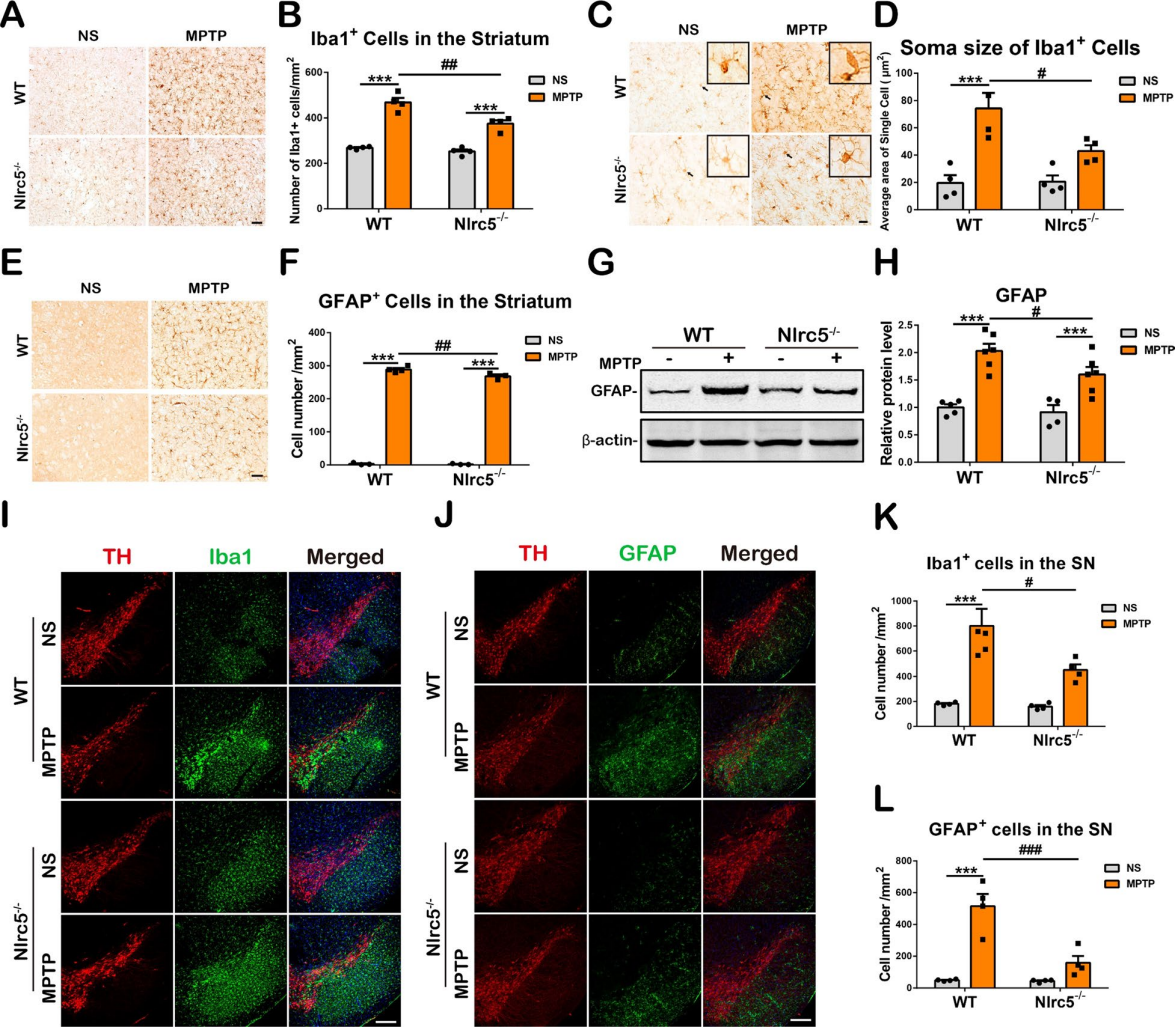
Assessments of glial activation in the nigrostriatal pathway of WT and *Nlrc5*^*−/−*^ mice at 7 days after MPTP administration. **A**, **B** Immunohistochemical staining and counting of Iba1^+^ cells in the striatum. Scale bar, 50 μm. **C**, **D** Soma size of Iba1^+^ cells in the striatum. Scale bar, 20 μm. **E**, **F** Immunohistochemical staining and counting of GFAP^+^ cells in the striatum. Scale bar, 50 μm. **G**, **H** GFAP protein levels in the striatum detected by Western-Blot. **I** Immunofluorescence double staining of TH (red) and Iba1 (green) in the substantia nigra. Scale bar, 200 μm. **J** Immunofluorescence double staining of TH (red) and GFAP (green) in the substantia nigra. Scale bar, 200 μm. **K** Counting of Iba1^+^ cells in the SNpc. **L** Counting of GFAP^+^ cells in the SNpc. All data were presented as the means ± SEM. n = 4. ^#^*p* < 0.05, ^##^*p* < 0.01, and *** or ^###^*p* < 0.001

Microglia and astrocytes are two crucial cell types that regulate neuroinflammation. Abnormal and persistent activation of microglia and astrocytes takes place in PD patients and PD animals, which contributes to the death of DA neurons [43]. Microglia, which are the principal innate immune cells in the CNS, can be assessed by Iba1-immunoreactive staining. Seven days after MPTP administration, microglial cells were significantly activated in the striatum, as indicated by an increased number of Iba1^+^ cells, extended cell-body size, an increase in bulging branches and higher cell ellipticity, and these activation features were milder in *Nlrc5*^−/−^mice (Fig. 4A–D). CD16 is a microglial M1 activation marker [44, 45]. The CD16^+^/Iba1^+^ ratio was elevated in the striatum of MPTP-induced PD mice, and this ratio was significantly reduced in *Nlrc5*^−/−^ mice compared to WT controls (Additional file 1: Fig. S3B, C). Likewise, dramatically increased numbers of MPTP-induced activated microglial cells were detected in the SNpc of WT mice but not in mice with *Nlrc5* deficiency (Fig. 4I, K). Moreover, astrocytes were assessed by GFAP-immunoreactive staining, and low astrocytic density in the striatum and the SNpc regions was detected in the NS groups. Seven days after MPTP administration, more intensely activated features of astrocytes, including an increased number of GFAP^+^ cells and extended cell-body sizes, were observed in the striatum and the SNpc of WT mice compared to *Nlrc5*^−/−^ mice (Fig. 4E, F, J, L). In addition, reduced GFAP protein levels were detected in the striatum of *Nlrc5*^−/−^mice by immunoblot analysis (Fig. 4G, H). These results demonstrated that *Nlrc5* deficiency reduced microglia and astrocyte activation in the nigrostriatal axis of MPTP-treated mice.

### *Nlrc5* deficiency alters the expression of inflammatory molecules in the striatum

**Fig. 5:**
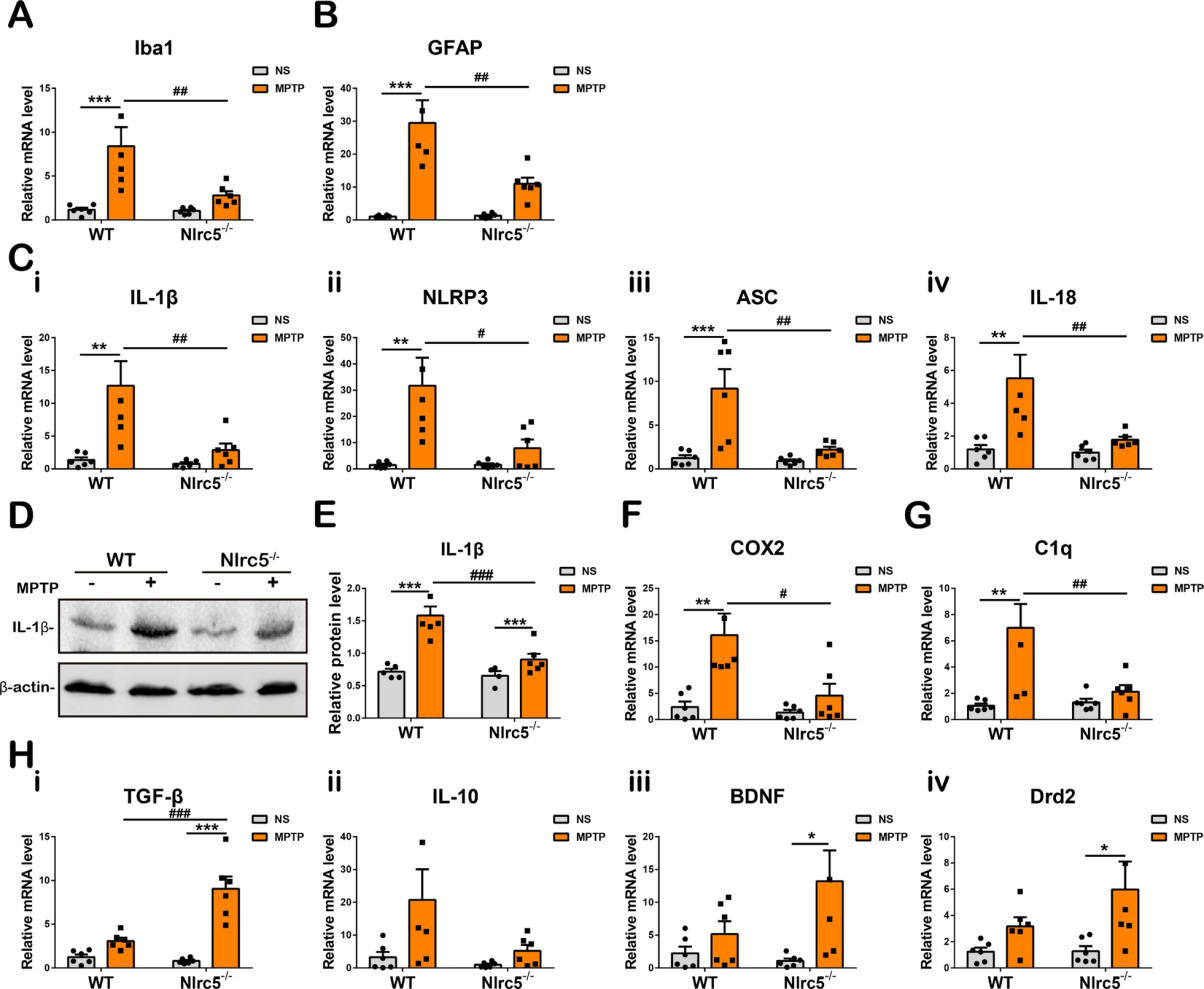
The expression of inflammatory molecules in the striatum of WT and *Nlrc5*^*−/−*^ mice at 3 days after MPTP administration. **A**, **B** Transcriptions of *Iba1* and *Gfap* in the striatum. **C** Transcriptions of *IL-1β* (**i**), *NLRP3* (**ii**), *ASC* (**iii**) and *IL-18* (**iv**). **D**, **E** Protein levels of IL-1β in the striatum detected by Western-Blot. **F**, **G** Transcriptions of *COX2* and *C1q* in the striatum. **H** The expression of anti-inflammatory molecules including *TGF-β* (**i**), *IL-10* (**ii**), *BDNF* (**iii**) and *Drd2* (**iv**) detected by qPCR. All data were presented as the means ± SEM. *n* = 4–6. * or ^#^*p* < 0.05, ** or ^##^*p* < 0.01, and *** or ^###^ *p* < 0.001

Furthermore, the expression of inflammatory molecules in the striatum was investigated. Three days after MPTP administration, the transcription of *Iba1* and *Gfap* in WT mice were markedly higher than those in NS-treated WT controls and MPTP-treated *Nlrc5*^*−/−*^ mice. *Iba1* and *Gfap* transcription levels were not changed in *Nlrc5*^*−/−*^ mice after MPTP administration (Fig. 5A, B). Interleukin-1β (IL-1β) and the inflammasome molecules NOD-like receptor protein 3 (NLRP3) and apoptosis-associated speck-like protein (ASC) are upregulated in MPTP-induced PD models and then impair DA neurons [46]. Our results demonstrated that the transcription of *IL-1β*, *NLRP3*, *ASC* and *IL-18* was increased in the striatum of WT mice, while *Nlrc5* deficiency significantly inhibited the upregulation of these four genes (Fig. 5C). Meanwhile, immunoblotting showed that the striatal protein levels of IL-1β were reduced by *Nlrc5* deficiency 3 days after MPTP administration (Fig. 5D, E). Moreover, reduced transcription of the proinflammatory molecules *COX2* and *C1q* was observed in the striatum of *Nlrc5*^*−/−*^ mice (Fig. 5F, G). On the other hand, the transcription of anti-inflammatory molecules, including *TGF-β* and *IL-10,* and neuroprotective molecules, such as *BDNF* and *Drd2*, were not altered in WT mice, while *Nlrc5* deficiency significantly increased the transcription of *TGF-β*, *BDNF* and *Drd2* but not *IL-10*. Moreover, the expression of *TGF-β* was markedly elevated in KO mice compared with WT controls after MPTP injection (Fig. 5H). These results suggested that *Nlrc5* deficiency effectively inhibited the expression of proinflammatory molecules in the striatum and altered the expression of anti-inflammatory and neuroprotective molecules after MPTP administration.

### The expression of proinflammatory molecules is suppressed in glial cells with NLRC5 deficiency

**Fig. 6:**
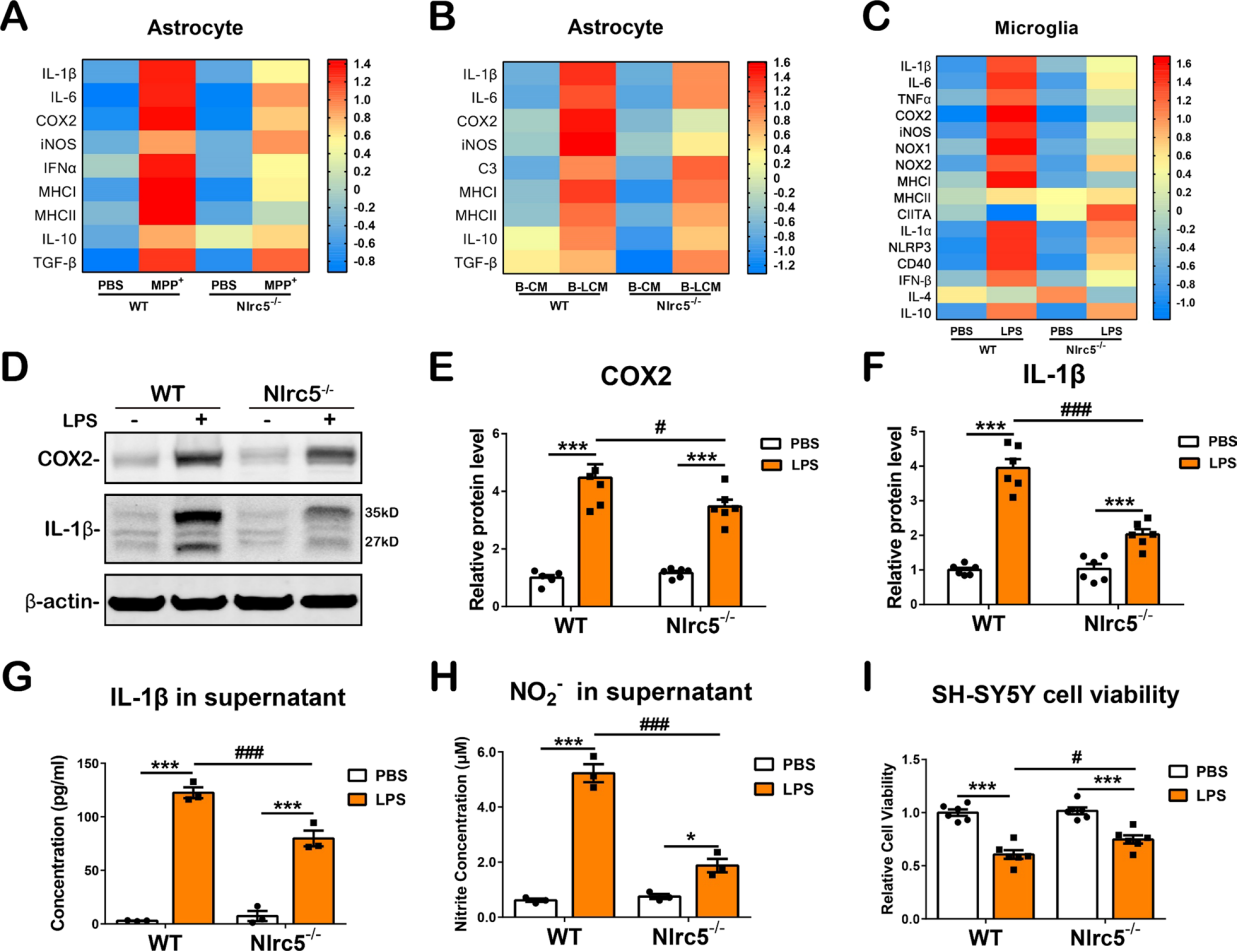
Inflammatory molecules expression in astrocytes, microglia and mixed glial cells treated with neuroinflammatory stimuli. **A** Transcriptions of *IL-1β, IL-6, COX2, iNOS, IFN-α, MHC I, MHC II, IL-10* and *TGF-β* in enhanced primary astrocytes culture detected by RT-qPCR 24 h after 1 mM MPP^+^ stimulation. n = 3–6. **B** Transcriptions of *IL-1β, IL-6, COX2, iNOS, C3, MHC I, MHC II, IL-10* and *TGF-β* in enhanced primary astrocytes culture detected by RT-qPCR 24 h after LPS-induced BV2 conditioned medium (B-LCM) stimulation. *n* = 4. **C** Transcriptions of *IL-1β, IL-6, TNF-α, COX2, iNOS, NOX1, NOX2, MHC I, MHC II, CIITA, IL-1α, NLRP3, CD40, IFN-β, IL-4* and *IL-10* in enhanced primary microglia culture detected by RT-qPCR 6 h after 100 ng/mL LPS stimulation. n = 3–7. **D–F** Protein levels of COX2 and IL-1β detected by immunoblotting. **G** Concentration of IL-1β proteins in the supernatants of mixed glial cells measured by ELISA. **H** Concentration of NO_2_^−^ in the supernatants of mixed glial cells. **I** Cell viability of SH-SY5Y cells treated with the supernatants from PBS- or LPS-stimulated WT or *Nlrc5*^*−/−*^ mixed glial cells. All data were presented as the means ± SEM. *n* = 3–6. ^#^*p* < 0.05, ** or ^##^*p* < 0.01, *** or ^###^*p* < 0.001

Through the upregulation of proinflammatory cytokines and molecules, activated microglia and astrocytes contribute to neuronal degeneration [3, 5]. Here, the inflammatory responses of these two glial cells were further investigated in enriched primary cell cultures in vitro. MPP^+^ (1 mM) was used to challenge primary astrocytes and initiate toxic conditions in MPTP-induced PD models [47, 48]. MPP^+^ treatment significantly increased the transcription of proinflammatory cytokines, including *IL-1β*, *IL-6*, *COX2*, *iNOS*, *IFN-α*, *MHC I* and *MHC II*, and this increase was abrogated in *Nlrc5*-deficient astrocytes (Fig. 6A). Our previous study showed that the supernatant from the activated murine microglial cell line BV-2 contained proinflammatory molecules (IL-1β, IL-6, TNF-α and NO) and could induce robust activation in primary astrocytes, which simulated immune regulation between microglia and astrocytes [13]. LPS-induced BV-2 conditioned medium (B-LCM) upregulated *IL-1β*, *IL-6*, *COX-2*, *iNOS*, *complement 3* (C3) and *MHC I* expression in WT astrocytes, whereas the expression of *IL-6*, *COX2* and *MHC I* was reduced in *Nlrc5*^*−/−*^ astrocytes, and the expression levels of *MHC II*, *IL-10*, and *TGF-β* showed no significant differences between the groups (Fig. 6B). Enriched WT and *Nlrc5*^*−/−*^ microglial cells were challenged with 100 ng/mL LPS to address whether *Nlrc5* deficiency affected microglial activation. *Nlrc5* deficiency significantly abrogated the increases in typical proinflammatory cytokines, including *IL-1β*, *IL-6*, *TNF-α*, *COX2*, *iNOS*, *NOX1*, *NOX2*, *IL-1α*, *NLRP3* and *IFN-β*, as well as the marker of activated microglia, *CD40*. Notably, *MHC I,* which is a target gene of NLRC5, was decreased in *Nlrc5*^*−/−*^ microglia in the resting state and was obviously suppressed in *Nlrc5*^*−/−*^ microglia compared to WT microglia after LPS stimulation (Fig. 6C). However, *Nlrc5* deficiency had no significant effect on the expression of the anti-inflammatory molecules *IL-4* and *IL-10* in microglia (Fig. 6C). Furthermore, the comprehensive inflammatory response of glial cells was investigated in mixed glial cultures (mainly composed of astrocytes and microglia) exposed to 250 ng/mL LPS. Immunoblotting showed that LPS significantly upregulated COX2 and pro-IL-1β protein levels in glial cells with the two genotypes; however, the levels of these two proinflammatory proteins were dramatically reduced in *Nlrc5*-deficient glial cells (Fig. 6D–F). Moreover, the levels of secreted IL-1β and NO in the supernatant were also suppressed in *Nlrc5*^*−/−*^ glial cell cultures (Fig. 6G, H). In addition, the supernatant of mixed glial cells challenged with LPS or PBS was incubated with SH-SY5Y cells, and cell viability was analyzed. WT glial supernatant exacerbated the degeneration of SH-SY5Y cells compared to the supernatant of *Nlrc5*^*−/−*^ glial cells (Fig. 6I). Likewise, MPP^+^ treatment for 24 h induced considerable COX2 protein expression in WT glial cells, and *Nlrc5* deficiency significantly attenuated the protein expression of COX2, as detected by immunoblotting (Additional file 1: Fig. S4A, B). These results suggested that *Nlrc5* deficiency suppressed the activation of microglia and astrocytes and inhibited the expression of proinflammatory factors.

### *Nlrc5* deficiency alters the activation of NF-κB and other inflammation-related signaling pathways in mixed glial cells

**Fig. 7:**
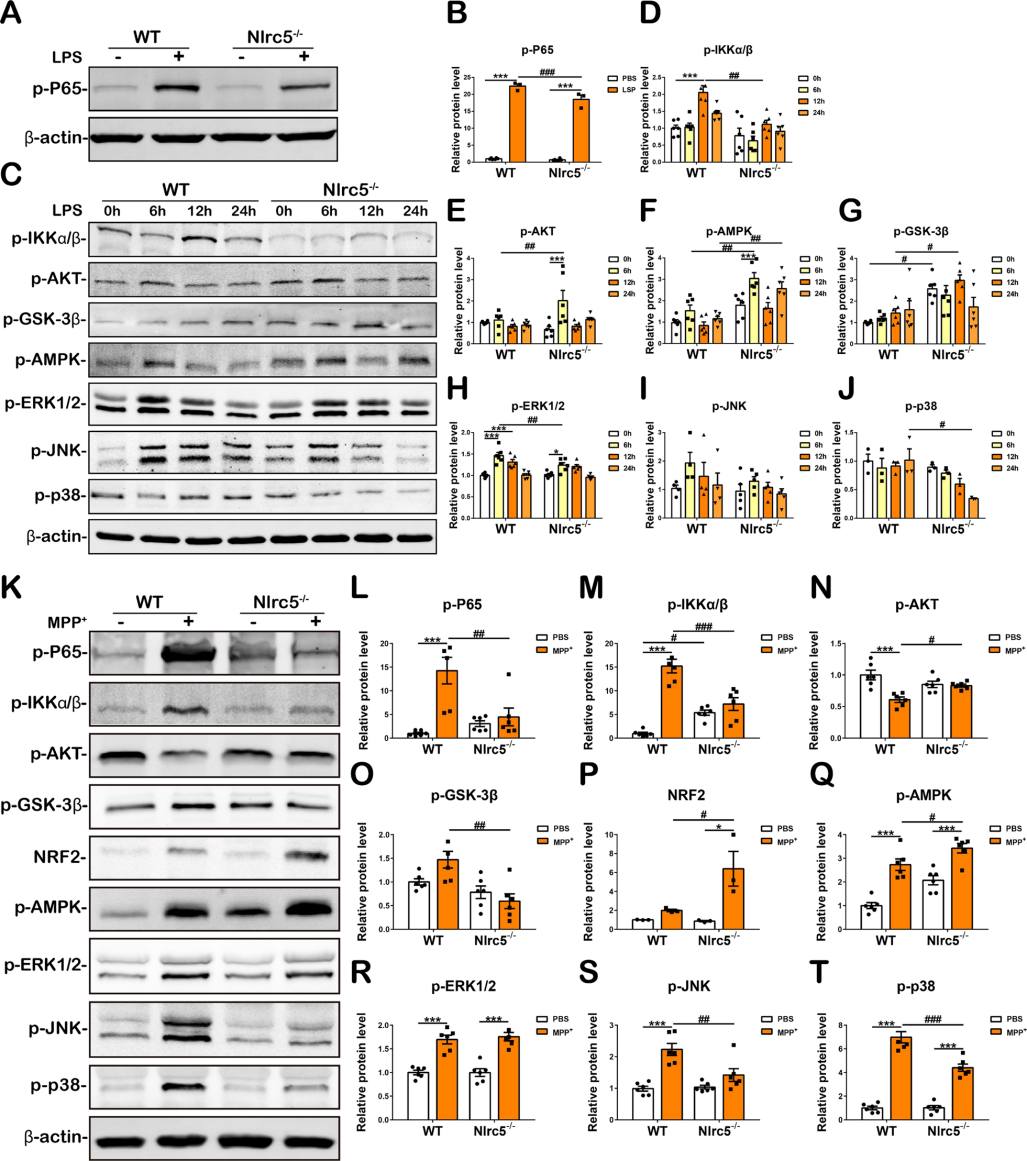
Analysis of NF-κB, AKT–GSK3β, AMPK and MAPK signaling pathways in mixed glial cells treated with neuroinflammatory stimuli. (**A**, **B** Phosphorylation of NF-κB P65 protein in mixed glial cells detected by immunoblotting 24 h after 250 ng/mL LPS stimulation. **C–J** After treatment with 250 ng/mL LPS for 0 h, 6 h, 12 h, 24 h, the protein levels of p- IKKα/β (**C**, **D**), p-AKT (**C**, **E**), p- AMPK (**C**, **F**), p- GSK3β (**C**, **G**), p-ERK1/2 (**C**, **H**), p-JNK (**C**, **I**) and p-p38 (**C**, **J**) detected by immunoblotting. *n* = 6. **K–T** After treatment with 1 mM MPP^+^ for 24 h, the protein levels of p-P65 (**K**, **L**), p-IKKα/β (**K**, **M**), p-AKT (**K**, **N**), p-GSK-3β (**K**, **O**), NRF2 (**K**, **P**), p-AMPK (**K**, **Q**), p-ERK1/2 (**K**, **R**), p-JNK (**K**, **S**) and p–p38 (**K**, **T**) were detected by Immunoblotting analysis. *n* = 3–6. All data were presented as the means ± SEM. * or ^**#**^*p* < 0.05, ** or ^##^*p* < 0.01, *** or ^###^*p* < 0.001

To further investigate the mechanism of the reduced inflammatory response in *Nlrc5-*deficient glial cells, mixed glial cells were treated with LPS (250 ng/mL) or MPP^+^ (1 mM), and inflammation-related signaling pathways were investigated by immunoblot analysis. Activation of the NF-κB pathway can be indicated by the phosphorylation of the P65 subunit and its upstream regulator IKKα/β. In WT mixed glial cells, the protein levels of phosphorylated P65 (p-P65) and phosphorylated IKKα/β (p-IKKα/β) were upregulated at 24 h and 12 h, respectively, after LPS stimulation, but the phosphorylation of P65 and IKKα/β was largely reduced in *Nlrc5*^*−/−*^ mixed glia (Fig. 7A–D). The AKT signaling pathway and its downstream molecule GSK-3β participate in the regulation of neuroglial cell activation and neuroinflammation [49], and AMPK plays an important role in preventing NF-κB activation and neuroprotection [50, 51]. The immunoblot results demonstrated that LPS stimulation barely induced the phosphorylation of AKT, GSK-3β and AMPK in WT mixed glial cells; However, *Nlrc5*^−/−^ mixed glial cells exhibited enhanced phosphorylation of these three proteins, but with different patterns. At 6 h after LPS treatment, phosphorylated AKT (p-AKT) and phosphorylated AMPK (p-AMPK) were markedly increased in *Nlrc5*-deficient glial cells compared to their WT counterparts and PBS-treated *Nlrc5*^−/−^ glial cells. At 24 h after LPS treatment, p-AMPK remained increased in *Nlrc5*-deficient glial cells compared to their WT counterparts. At baseline and 12 h after LPS treatment, phosphorylated GSK-3β (p-GSK-3β) was increased in *Nlrc5*-deficient glial cells (Fig. 7C, E–G). MAPK signaling includes three main transduction pathways (ERK1/2, JNK and p38), which regulate neuroinflammation and glial cell polarization [52, 53]. Phosphorylation of ERK1/2 (p-ERK1/2) was induced by LPS at 6 h and 12 h in WT mixed glial cells, and this effect was suppressed by *Nlrc5* deficiency, while phosphorylated JNK (p-JNK) and phosphorylated p38 (p–p38) remained unchanged in the different groups of glial cells. Notably, the p–p38 protein levels in *Nlrc5-*deficient glia were significantly lower than those in their WT counterparts 24 h after LPS stimulation (Fig. 7C, H–J).

In addition, neuroinflammation-related signaling pathways were examined in mixed glial cells treated with MPP^+^. Immunoblotting showed that the phosphorylation of P65 and IKKα/β was increased in WT mixed glia, whereas *Nlrc5* deficiency dramatically suppressed the expression of p-P65 and p-IKKα/β 24 h after MPP^+^ stimulation (Fig. 7K–M). Nuclear factor E2-related factor 2 (NRF2) is a downstream target of the AKT signaling pathway and acts as a negative regulator of NF-κB during PD-associated neuroinflammation [54, 55]. MPP^+^ stimulation decreased the phosphorylation of AKT in WT mixed glial cells, while p-AKT levels in MPP^+^-treated *Nlrc5*-deficient glial cells were not altered and were significantly higher than those in their WT counterparts (Fig. 7K, N). Moreover, *Nlrc5* deficiency significantly reduced the phosphorylation of GSK-3β after MPP^+^ treatment (Fig. 7K, O). However, the protein levels of NRF2 and p-AMPK in *Nlrc5*^*−/−*^ mixed glial cells were dramatically higher than those in their WT counterparts after MPP^+^ treatment (Fig. 7K, P, Q). MAPK signaling pathways in glial cells of two genotypes were activated 24 h after MPP^+^ treatment, except p-JNK was only increased in WT glial cells, whereas *Nlrc5* deficiency largely reduced the protein levels of p-JNK and p–p38 but had no effects on p-ERK1/2 after MPP^+^ stimulation (Fig. 7K and R-T). Overall, *Nlrc5* deficiency effectively suppressed the activation of the NF-κB signaling pathway, promoted the activation of the AKT and AMPK pathways and the downstream molecules GSK-3β and NRF2, and partially inhibited the activation of MAPK signaling pathways in mixed glial cells treated with LPS or MPP^+^.

### ***Nlrc5*** deficiency mitigates MPP^+^- and LCM-induced neuronal death

**Fig. 8:**
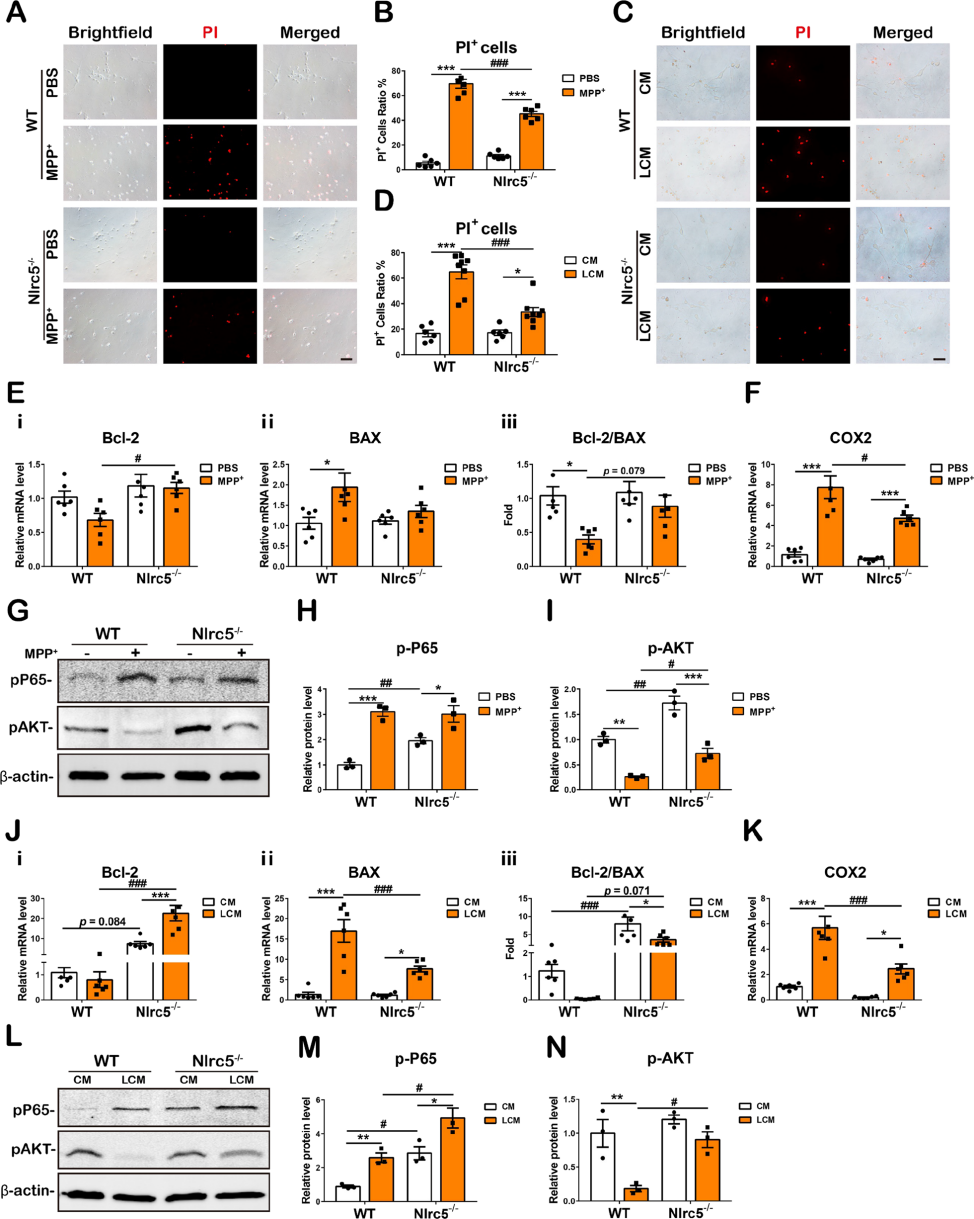
MPP^+^ and LCM-induced cell death and neuronal survival-related molecule expression in primary neurons. **A** Representative PI staining and imaging of primary neurons treated with 20 μM MPP^+^ for 24 h. Scale bar, 50 μm. **B** Counting and statistical analyses of PI^+^ neurons. **C** Representative PI staining and imaging of primary neurons treated with CM or LCM for 24 h. Scale bar, 50 μm. **D** Counting and statistical analyses of PI^+^ neurons. **E** Transcriptions of apoptosis-related molecules *Bcl-2* (i), *BAX* (**ii**) detected by RT-qPCR and ratio of *Bcl2/BAX* (**iii**) in primary neurons treated with 20 μM MPP^+^ for 24 h. **F** Transcriptions of oxidative stress-related molecules *COX2*. **G–I** Protein levels of p-P65 and p-AKT in primary neurons treated with 20 μM MPP^+^ for 24 h detected by immunoblotting. **J** Transcriptions of apoptosis-related molecules *Bcl-2* (**i**), *BAX *(**ii**) detected by RT-qPCR and ratio of *Bcl2/BAX* (**iii**) in primary neurons treated with CM or LCM for 24 h. **L–N** Protein levels of p-P65 and p-AKT in primary neurons treated with CM or LCM for 24 h detected by immunoblotting. All data were presented as the means ± SEM. *n* = 3–6. * or ^#^*p* < 0.05, ** or ^##^*p* < 0.01, and *** or ^###^*p* < 0.001

Furthermore, to confirm whether *Nlrc5* deficiency had a direct effect on neuronal survival in neurotoxic conditions, PBS or MPP^+^ (20 μM) and the conditioned medium from PBS- or LPS-induced mixed glial cells (CM/LCM) were used to challenge the two genotypes of primary neurons. After 24 h, dead neurons were analyzed by staining with propidium iodide (PI), a DNA-specific red fluorescent dye that is permeant only to dead cells. Statistical analyses revealed that MPP^+^ caused dramatic death in WT and *Nlrc5*^*−/−*^ neurons; however, neuronal death was attenuated by *Nlrc5* deficiency (Fig. 8A, B). LPS could effectively induce M1 polarization in microglia, which subsequently induced neurotoxic reactive A1-type astrocytes, which release enormous amounts of proinflammatory molecules into the medium. LCM significantly induced neuronal death at 24 h, and *Nlrc5*^*−/−*^ neurons were less vulnerable to LCM than their WT counterparts (Fig. 8C, D). Furthermore, the expression of apoptosis- and oxidative stress-related genes in neurons was assessed. MPP^+^ treatment did not change the transcription of the antiapoptotic gene *Bcl-2* in WT or *Nlrc5*^−/−^ neurons; however, *Bcl-2* transcription in *Nlrc5*^−/−^ neurons was markedly higher than that in WT neurons after MPP^+^ treatment. A significant increase in the proapoptotic gene *Bax* and an obvious decrease in the *Bcl-2* to *Bax* ratio were detected in WT neurons but not in *Nlrc5*^*−/−*^ neurons [Fig. 8E(i–iii)]. In MPP^+^-treated neurons, the expression of *COX2* was dramatically upregulated, while *Nlrc5* deficiency largely reduced the expression of *COX2* (Fig. 8F). In addition, activation of the neuronal survival-related signaling pathways NF-κB and AKT was investigated. *Nlrc5*^*−/−*^ neurons exhibited higher p-P65 expression than WT neurons at baseline, and WT and *Nlrc5*^*−/−*^ neurons showed comparatively higher p-P65 expression after MPP^+^ treatment (Fig. 8G, H). MPP^+^ treatment prominently decreased the phosphorylation of AKT in neurons of two genotypes, but p-AKT levels in *Nlrc5*^*−/−*^ neurons were significantly higher than those in their WT counterparts at baseline and after MPP^+^ treatment (Fig. 8G, I). In CM- and LCM-treated WT neurons, *Bcl-2* transcription was not changed, while *BAX* expression was increased after LCM treatment. *Nlrc5* deficiency increased the transcription level of *Bcl-2* in LCM-treated neurons compared to CM-treated neurons and their LCM-treated WT counterparts. Furthermore, *Nlrc5* deficiency increased *BAX* expression in LCM-treated neurons compared to CM-treated neurons; however, the expression of *BAX* was significantly lower in *Nlrc5*^*−/−*^ neurons than in their WT counterparts after LCM treatment, and *Nlrc5*^*−/−*^ neurons exhibited a much higher *Bcl-2*/*BAX* ratio than WT neurons at baseline (Fig. 8J). Similarly, the expression levels of *COX2* were induced in both genotypes of neurons after LCM treatment, and *Nlrc5* deficiency significantly abrogated the increase in *COX2* transcription (Fig. 8K). Likewise, LCM treatment increased p-P65 expression and decreased p-AKT expression in WT neurons. In *Nlrc5*^*−/−*^ neurons, the phosphorylation of P65 was higher at baseline than in their WT counterparts and was increased further after LCM treatment, while p-AKT was not altered with LCM treatment. p-P65 and p-AKT protein levels were higher in LCM-treated *Nlrc5*^*−/−*^ neurons than in LCM-treated WT controls (Fig. 8L–N). Collectively, neurons with *Nlrc5* deficiency exhibited resistance to MPP^+^ or LCM, and the underlying mechanism might be associated with increases in the phosphorylation of NF-κB P65 and AKT and prosurvival gene expression.

### The expression of NLRC5 and immune-related genes in the peripheral blood of healthy subjects and PD patients

**Fig. 9:**
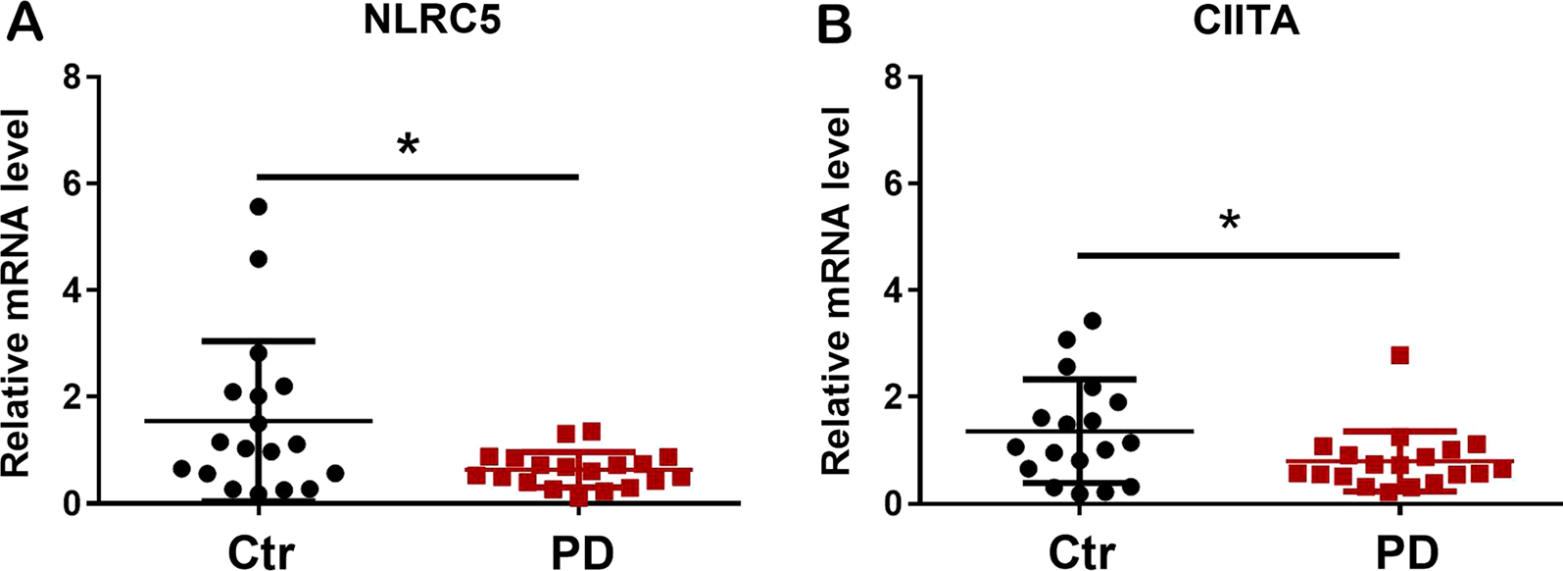
Transcriptions of *NRLC5* and immune-related genes in the peripheral blood of healthy subjects and PD patients. Transcriptions of *NLRC5* (**A**) and *CIITA* (**B**) detected by RT-qPCR. All data were presented as the means ± SEM. *n* = 18–19. *p < 0.05

Our results showed that the expression of NLRC5 was inducible in PD mice and PD cell models. Therefore, to examine the expression of NLRC5 in the peripheral blood of PD patients and whether NLRC5 could be a potential biomarker for PD, the transcription of *NLRC5* and related genes was measured in the whole peripheral blood of healthy subjects (Ctr) and patients with PD. The RT‒qPCR results demonstrated that the transcription of *NLRC5* and class II major histocompatibility complex transactivator (*CIITA*) was decreased (Fig. 9A, B) in the blood of PD patients. In the receiver-operating characteristic curve analysis, the value of area under curve (AUC) was 0.69 for NLRC5 (sensitivity 60%, specificity 82%), 0.68 for CIITA (sensitivity 60%, specificity 81%) (Additional file 1: Fig. S5).

## Discussion

NLRC5, which is a key transcriptional activator of MHC I and regulator of the NF-κB pathway, has been shown to play a crucial role in peripheral immune responses [21, 22]. Many neurodegenerative diseases, including PD, are closely associated with the innate immune response and inflammation [7]. To date, studies of the function of NLRC5 in the CNS are still rare. In this study, we demonstrated that *Nlrc5* deficiency attenuated PD pathological characteristics, such as the loss of dopaminergic neurons, damage to the DA system, motor deficits and glial cell activation, in MPTP-induced PD mice. Suppressed activation of the NF-κB pathway and reduced production of proinflammatory molecules were further observed in *Nlrc5*-deficient glial cells, and the effect of the *Nlrc*5 mutation on survival was observed in neurons.

Accumulating evidence suggests that neuroinflammation is an important pathology and marker of the progression of PD, which is mediated by microglia and astrocytes [56, 57]. Due to the activation of microglia and astrocytes, the levels of proinflammatory molecules, including IL-1β, IL-6, TNF-α, iNOS and ROS, are elevated in the nigrostriatal system of postmortem human samples, increasing the risk of dopaminergic neuronal degeneration [5, 58]. As a consequence, regulating neuroinflammation by reducing glial activation might be a potential early therapeutic strategy for PD [43]. The results of our study showed that *Nlrc5* deficiency attenuated the activation of microglia and astrocytes in the nigrostriatal axis of MPTP-induced PD mice. Notably, many studies have suggested that microglia can shift to different polarization states in response to different stimuli or during different stages of inflammation [45]. The proinflammatory phenotype (M1) was assessed in the current study and was characterized by the significant upregulation of proinflammatory cytokines (including IL-1β, IL-6, TNF-α), iNOS, CD16 and the costimulatory molecule CD40 [59, 60]. These changes were reduced in *Nlrc5*^−/−^ mice after MPTP administration (Additional file 1: Fig. S3A, B) or in enriched mutant microglia challenged with LPS (Fig. 6C), indicating a reduction in proinflammatory polarization in microglia with *Nlrc5* deficiency, which was consistent with previous studies on the inflammatory responses of bone-marrow-derived or peritoneal macrophages in the periphery [30, 37]. In the inflammatory context of PD, astrocytes can be converted into a neurotoxic phenotype (A1) by activated microglia, and these cells release proinflammatory cytokines and ROS [61, 62]. Similar to microglia, reactive astrocytes were alleviated by *Nlrc5* deficiency in the nigrostriatal axis, as determined by GFAP-immunoreactive staining and immunoblot analysis (Fig. 4E–H, J, L). Next, suppressed expression of proinflammatory molecules was observed in enriched *Nlrc5*^*−/−*^astrocytes challenged with MPP^+^ and B-LCM (Fig. 6A, B). In this context, astrocytes may amplify neuroinflammation. In MPTP-treated *Nlrc5*^*−/−*^ mice, weaker microglial activation led to fewer reactive astrocytes with reduced proinflammatory feedback, resulting in a decrease in overall inflammation levels in the striatum after MPTP administration (Fig. 5).

NF-κB is a core transcription factor associated with innate immune and inflammatory responses [63]. Nevertheless, the roles of NLRC5 in the NF-κB pathway are variable. NLRC5 has been reported to be a negative regulator of the NF-κB pathway by competing with the subunit NEMO to inhibit the phosphorylation of IKKα/β in some studies [20], whereas other studies pointed to the opposite conclusions [30]. In mixed glial cultures with *Nlrc5* deficiency, we demonstrated significant suppression of the phosphorylation of the NF-κB subunit P65 and IKKα/β in response to LPS and MPP^+^ stimulation (Fig. 7A–D, K–M), as well as reduced expression of the downstream genes *COX2* and *IL-1β* (Figs. 6D–F, S4). Crosstalk between other signaling pathway(s) and NF-κB regulates NF-κB signaling activity. Analysis of the striatal transcriptome of WT and *Nlrc5*^−/−^ mice was performed using the Kyoto Encyclopedia of Genes and Genomes (KEGG) and showed that the AKT, FoxO (downstream of AMPK), and MAPK signaling pathways were significantly enriched in *Nlrc5*^−/−^ mice (Additional file 1: Fig. S6). AKT–GSK-3β activation has an inhibitory effect on NF-κB [64], and the phosphorylation of AMPK suppresses the activation of NF-κB and neuroinflammation [65]. Deletion of AMPK increases IFN-γ-induced STAT1 activation and the immune response in glial cells [66], and an agonist of AMPK ameliorates microglial activation and the production of proinflammatory molecules, including IL-6, TNF-α, iNOS and COX-2 [67]. Notably, NRF2, an anti-inflammatory molecule induced by AKT and AMPK, negatively regulates the activation of NF-κB [68, 69], and its neuroprotective effects have been reported in PD [70]. In addition, the MAPK (mainly ERK, JNK and p38) signaling pathway plays a crucial regulatory role in neuroinflammation, inducing proinflammatory molecule expression in glial cells [71, 72]. Studies on BV2 cells have shown that the p38/MAPK pathway acts synergistically with NF-κB in response to LPS stimulation [73], and a p38 inhibitor significantly downregulates NF-κB activation and IL-6 expression [74]. In the current study, the phosphorylation of AKT and AMPK was increased in LPS- or MPP^+^-treated *Nlrc5*^−/−^ mixed glial cells compared to their WT counterparts (Fig. 7C, E, F), and NRF2 protein levels were elevated in *Nlrc5-*deficient glial cells (Fig. 7K, P). On the other hand, *Nlrc5* deficiency reduced the phosphorylation of p38 in LPS-treated mixed glial cells and significantly decreased the activation of JNK and p38 in mixed glial cells treated with MPP^+^, which were closely related to suppression of the glial inflammatory response [75]. Thus, we demonstrated that NF-κB activation in *Nlrc5*^−/−^ glial cells was suppressed as a consequence of the enhanced AKT–GSK-3β and AMPK activation and inhibition of the MAPK signaling pathway.

In PD patients and PD mouse models, the upregulation of IL-1β and IL-18 in the nigrostriatal axis induced by NLRP3 inflammasome activation damages dopaminergic neurons, whereas the suppression of inflammasome activation decreases IL-1β expression, thus attenuating PD-associated phenotypes[46, 76–78]. Increasing evidence indicates that the NLRC5 protein participates in the direct assembly and activation of inflammasomes and induces the secretion of IL-1β [79, 80]. In the present study, we demonstrated that the transcription of *IL-1β*, *NLRP3*, *ASC* and *IL-18* was significantly elevated in the striatum of mice after MPTP administration, and this effect was largely alleviated by *Nlrc5* deficiency (Fig. 5C–E). Meanwhile, the transcription of *IL-1β*, *NLRP3* and *IL-1α* was significantly reduced in LPS-induced *Nlrc5*^−/−^ microglia, as well as in LPS-induced mixed glial cells, compared to their WT counterparts (Fig. 6C, D, F, G). Therefore, these findings suggest that *Nlrc5* deficiency attenuates inflammasome activation and IL-1β expression in the striatum of MPTP-treated mice and LPS-induced glial cells, thereby contributing to neuroprotective outcomes. The underlying mechanism is worth exploring in depth.

DA neurons are vulnerable to glial activation, which is largely attributed to their expression of a wide range of cytokine and chemokine receptors [43]. As shown in recent work by Lee et al., neuronal viability was significantly reduced when the cells were cocultured with microglia treated with LPS [46]. A similar effect on SH-SY5Y cells was also reported in astrocyte cocultures treated with MPP^+^ [81]. In this study, significant neuronal cell death was observed in WT neurons treated with MPP^+^ and LCM, and this effect was largely ameliorated in *Nlrc5*^−/−^ neurons (Fig. 8A–D). These data prove that *Nlrc5* deficiency confers resistance to neurotoxicity in primary neurons.

Oxidative stress is known to accompany neuronal degeneration in the pathological progression of PD. The expression of COX2 was elevated in DA neurons after MPTP administration [82, 83], and an in vitro study revealed that MPP^+^ could directly induce oxidative stress in neurons and contribute to neuronal death [84]. We observed that MPP^+^ and LCM treatment significantly increased *COX2* transcription in neurons, and this effect was markedly inhibited by *Nlrc5* deficiency (Fig. 8F, K). A reduction in oxidative stress in *Nlrc5*^−/−^ neurons contributes to their resistance to neurotoxic stimulation.

Neuronal activation of NF-κB occurs in acute nerve injury and chronic neurodegenerative diseases, such as Alzheimer's disease (AD) and PD [85]. The NF-κB heterodimer p65/p50 upregulates the expression of the antiapoptotic molecule Bcl-2, while the NF-κB c-Rel homodimer directly induces the transcription of *Bcl-xL*, another apoptosis inhibitor [31]. Our previous study suggested that SH-SY5Y cells overexpressing NF-κB c-Rel exhibited significant resistance to MPP^+^ neurotoxicity [41]. Therefore, activation of NF-κB promotes the survival of neurons. As shown in Fig. 8G, H, L and M, *Nlrc5* deficiency increased the basal phosphorylation of NF-κB P65 in neurons, MPP^+^ and LCM significantly upregulated the levels of p-P65 in the two genotypes of neurons, and *Nlrc5* deficiency further promoted the phosphorylation of P65 in neurons treated with LCM. Although NLRC5 and NF-κB are closely associated, NLRC5 plays distinct roles in the regulation of NF-κB in glial cells and neurons. In conjunction with NF-κB, the AKT signaling pathway also plays a critical role in mediating neuronal cell survival [36, 86]. It has been reported that MPP^+^-induced apoptosis in dopaminergic neurons is accompanied by a decrease in AKT phosphorylation, while an increase in p-AKT has an antiapoptotic effect [87]. Similarly, we observed a decrease in p-AKT in MPP^+^/LCM-treated WT neurons, while *Nlrc5* deficiency dramatically increased the phosphorylation of AKT after stimulation (Fig. 8G, I, L and N). Intriguingly, conditioned medium (CM) from untreated mixed glial cells may contain balancing and supporting substances secreted by glial cells, which could explain why *Nlrc5* deficiency altered the basal phosphorylation of AKT in PBS-treated neurons; however, this difference was compromised in CM-treated neurons. In summary, *Nlrc5* deficiency leads to neuronal survival through enhanced activation of NF-κB and AKT and the inhibition of COX2 expression after neurotoxic treatment.

Recently, the concentration of NLRC5 was reported to be decreased in the serum of IgA nephritis (IgAN) patients and showed a negative correlation with pathological severity, while the expression of *NLRC5* was significantly increased in IgAN tissues [88]. Likewise, we observed a decrease in the transcription level of *NLRC5* in whole blood samples from PD patients. Based on the ROC curve analysis, NLRC5 might be a possible biomarker of PD (Additional file 1: Fig. S5). However, more samples are needed to support this conclusion. CIITA, another NLR family member, is structurally similar to NLRC5, and its crucial role in PD and neuroinflammation has gradually been revealed. Williams et al. reported that *CIITA* deficiency reduced α-syn-induced neurodegeneration [89]. In the current study, we also found that the transcription of *CIITA* was decreased in PD patient blood compared to that of healthy controls (Fig. 9B), which is consistent with an analysis of the profile from the GEO database (Additional file 1: Fig. S7). The underlying mechanism warrants further investigation.

## Conclusion

**Fig. 10:**
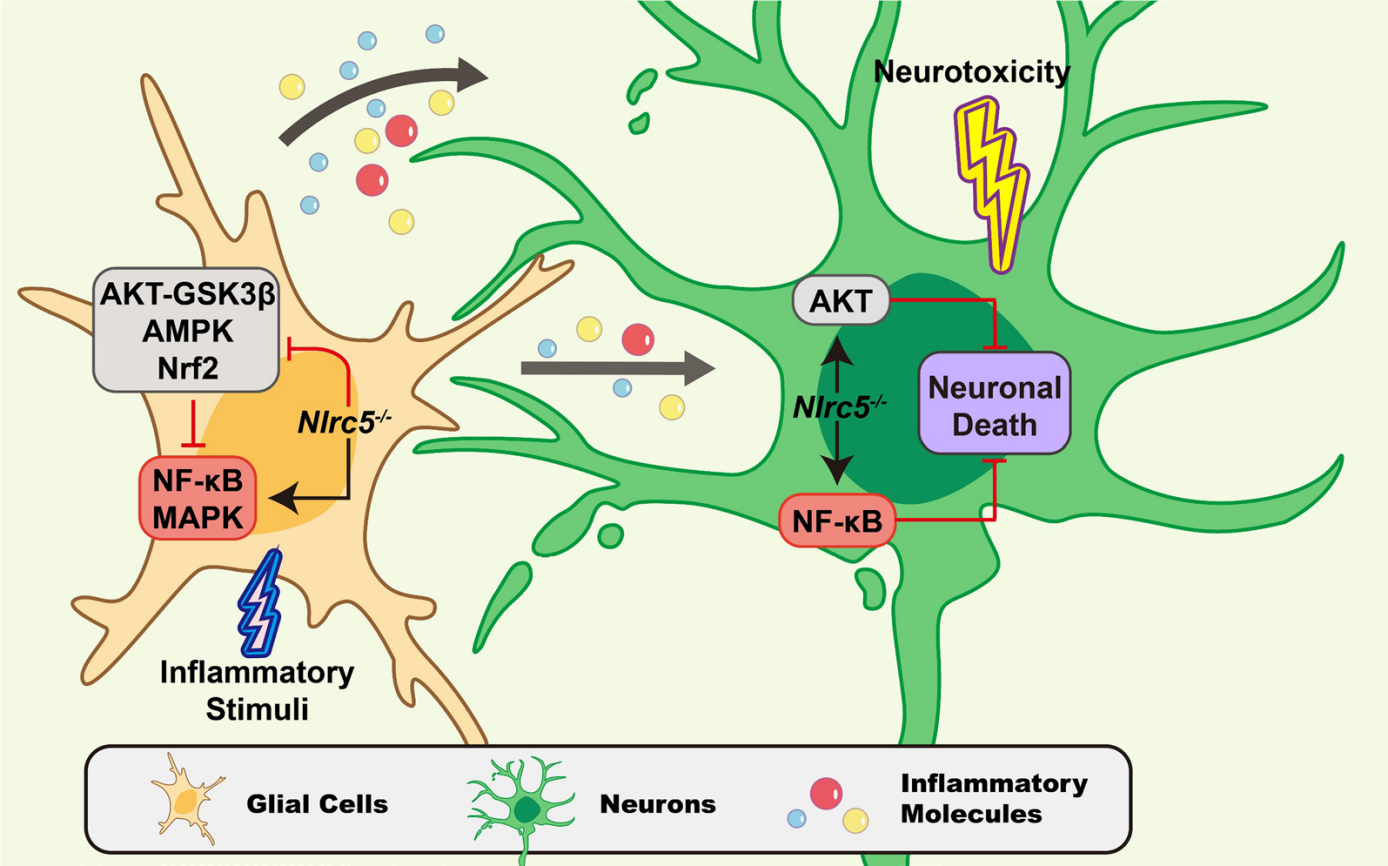
Diagram of *Nlrc5* in regulating neuroinflammation and neuronal survival. In glial cells the pro-inflammatory signalings NF-κB and MAPK are suppressed by *Nlrc5* deficiency, and AKT–GSK3β and AMPK pathways was enhanced. In neurons, *Nlrc5* deficiency causes upregulation of NF-κB and AKT, which promotes the survival of neurons

In the present study, we demonstrate that NLRC5 promotes neuroinflammation and dopaminergic degeneration in PD. *Nlrc5* deficiency attenuates glial activation by inhibiting the NF-κB and MAPK signaling pathways and reinforces neuronal protection by enhancing the activation of NF-κB and AKT in PD-related cell models (Fig. 10, Additional file 1: Fig. S8). The altered expression of NLRC5 in PD models suggests the emerging value of NLRC5 in regulating neuroinflammation.

## Supplementary Information

**Additional file 1.** Additional figures and Tables.

## Data Availability

The data and materials generated during the current study are not publicly available but are available from the corresponding author upon reasonable request.

## Abbreviations

AKT: Protein kinase B
AMPK: Adenosine 5′ Monophosphate (AMP)-activated protein kinase
BAX: Bcl-2-associated X protein
Bcl-2: B-cell lymphoma-2
BDNF: Brain-derived neurotrophic factor
C3: Complement 3
COX2: Cyclooxygenase type 2
DAPI: 4,6-Diamidino-2-phenylindole
DAT: Dopamine transporter
ERK: Extracellular signal-regulated kinase
GFAP: Glial fibrillary acidic protein
GSK-3β: Glycogen synthase kinase 3 beta
Iba1: Ionized calcium binding adapter molecule 1
IKK: I-kappa-B inhibitor kinases
IL: Interleukin
iNOS: Inducible nitric oxide synthase
JNK: C-Jun N-terminal kinase
LCM: LPS-stimulation conditional medium
LPS: Lipopolysaccharides
MAPK: Mitogen-activated protein kinase
MPP^+^: 1-Methyl-4-phenyl-pyridinium
MPTP: 1-Methyl-4-phenyl-1,2,3,6-tetrahydropyridine
NF-κB: Nuclear factor-kappa B
NOX: NADPH-oxidase
PD: Parkinson's disease
qPCR: Quantitative polymerase chain reaction
ROS: Reactive oxygen species
TGF-β: Transforming growth factor-β
TH: Tyrosine hydroxylase
TNF-α: Tumor necrosis factor-alpha
