# Supplementary material for: NOD-like receptor NLRC5 promotes neuroinflammation and inhibits neuronal survival in Parkinson’s disease models
Source: J Neuroinflammation. 2023 Apr 18;20:96. doi: 10.1186/s12974-023-02755-4 (PMC10111753; doi:10.1186/s12974-023-02755-4)
Supplement: Supplementary file 1 — Additional file 1. Additional figures and Tables. [file 12974_2023_2755_MOESM1_ESM.docx]

**Supplementary materials**

**NOD-like receptor NLRC5 promotes neuroinflammation and**

**Inhibits neuronal survival in Parkinson’s disease**

Zhaolin Liu, Chenye Shen, Heng Li, Jiabin Tong, Yufei Wu, Yuanyuan Ma, Jinghui Wang,

Zishan Wang, Qing Li, Xiaoshuang Zhang, Hongtian Dong, Yufang Yang, Mei Yu, Jian Wang, Renyuan Zhou, Jian Fei, Fang Huang

**Supplementary figures and legends**


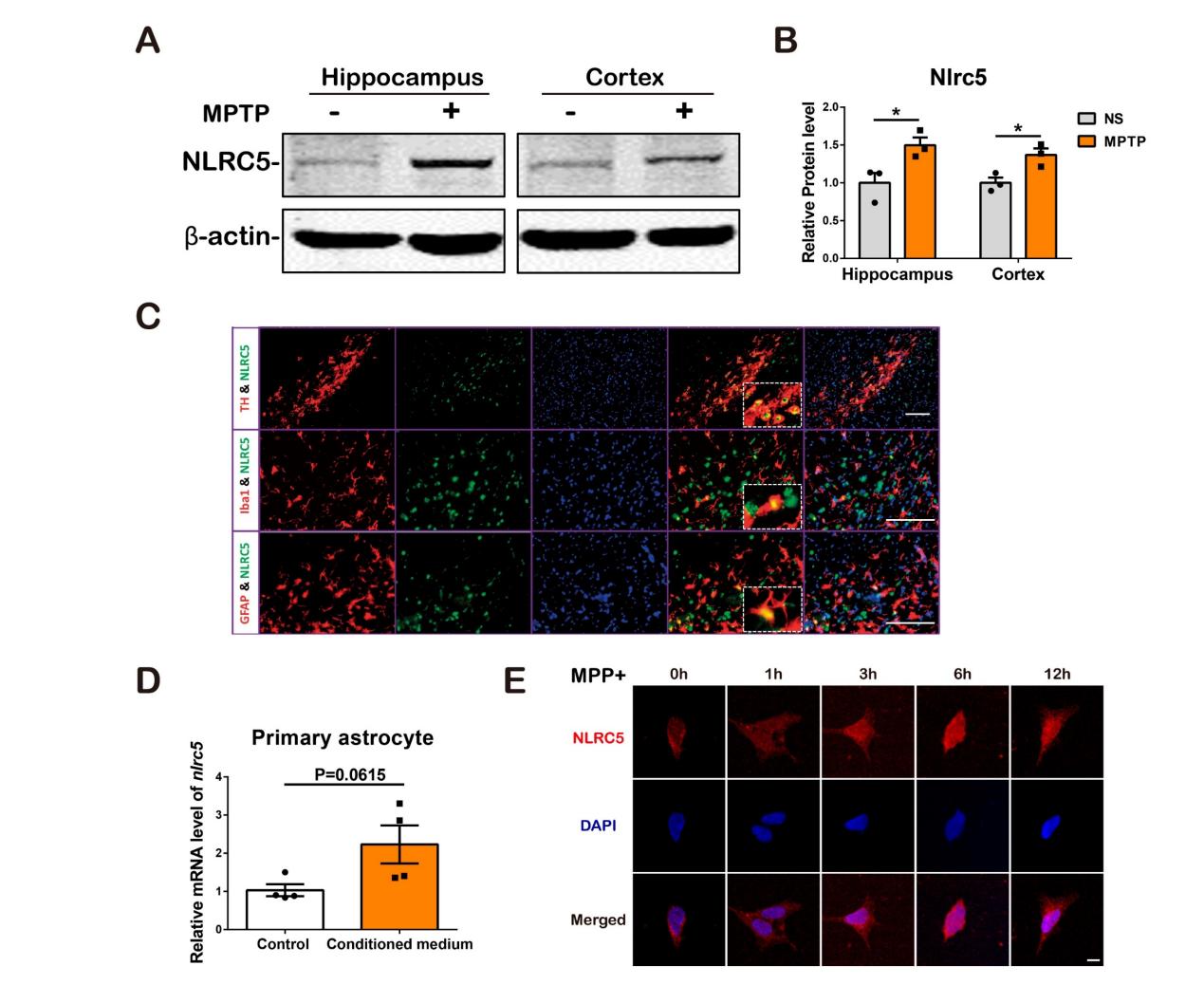


**Figure S1. The expression of NLRC5 in vivo and in vitro.** **(A, B)** Protein levels of NLRC5 in the hippocampus and the cortex detected by immunoblot at 7 days after NS or MPTP administration. n=3. * *p*<0.05. **(C)** Immunofluorescence double staining of TH (red) and NLRC5 (green) (top panel) or Iba1 (red) and NLRC5 (green) (middle panel) or GFAP (red) and NLRC5 (green) (bottom panel) in the substantia nigra. Scale bar, 100μm. **(D)** Transcriptions of *Nlrc5* in primary astrocytes treated with B-CM or B-LCM for 24h. n=4. **(E)** Immunocytochemistry staining of NLRC5 in SH-SY5Y cells treated with 1mM MPP^+^ for 0h, 1h, 3h, 6h, and 12h. Scale bar, 10 μm.


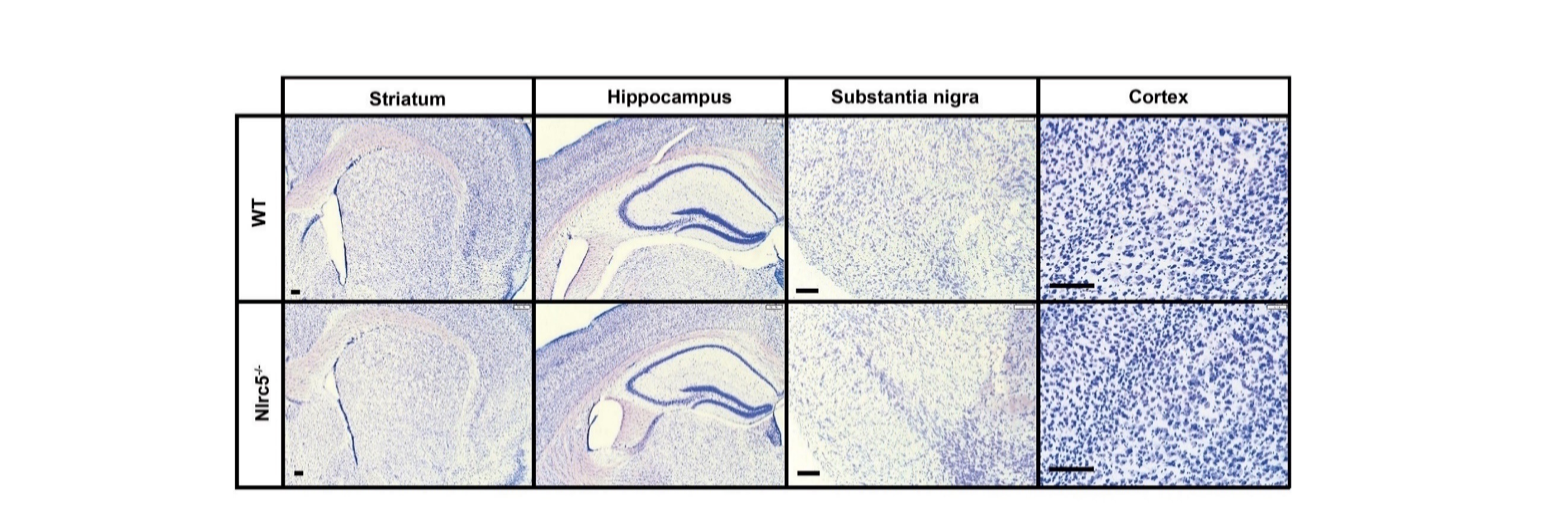


**Figure S2.** Nissl staining of the striatum, hippocampus, substantia nigra and cortex sections from WT and *Nlrc5^-/-^* mice. Scale bar, 100 μm.


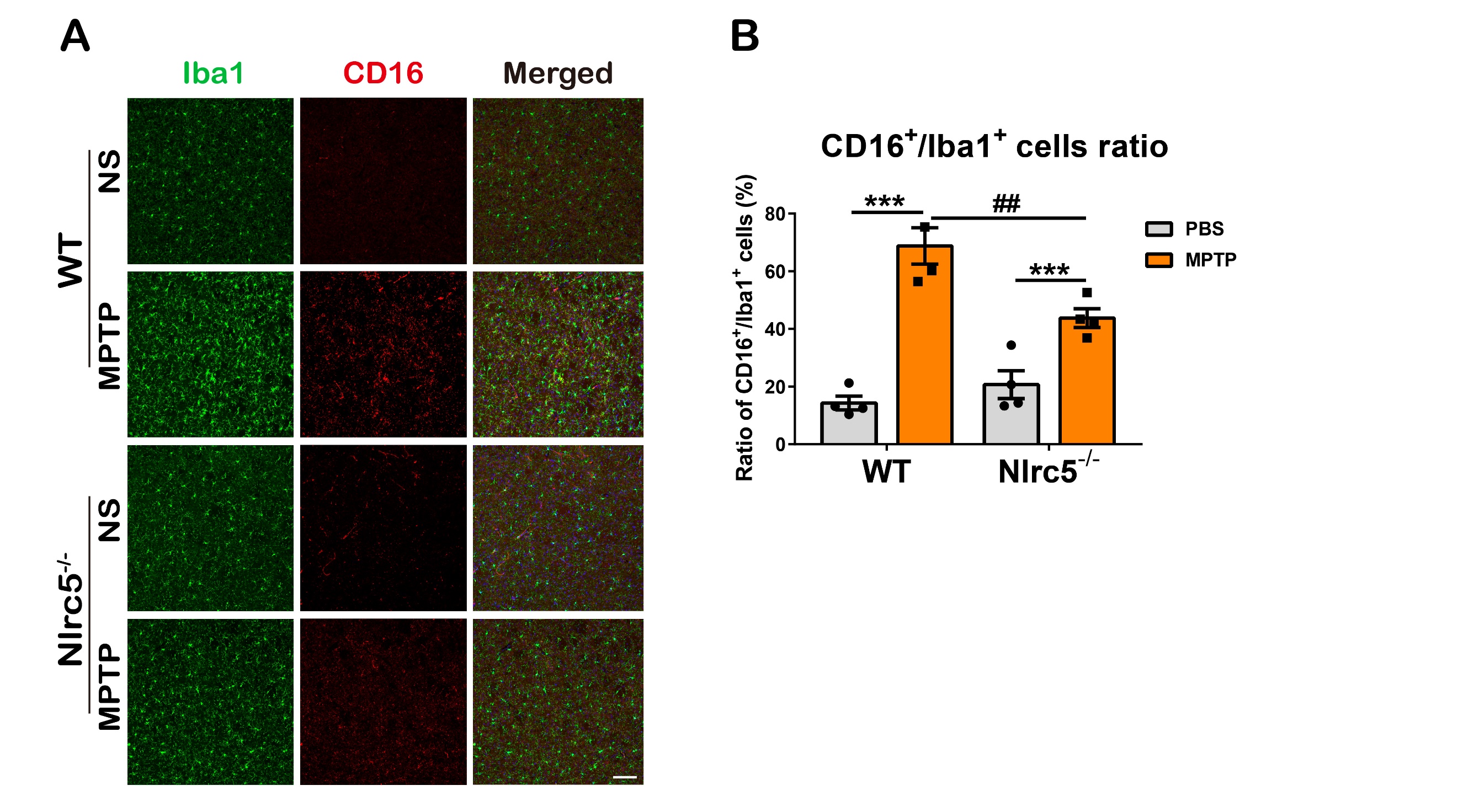


**Figure S3. The analysis of CD16^+^ M1 microglial cells in the striatum. (A)** Immunofluorescence double staining of Iba1 (green) and CD16 (red) in the striatum. Scale bar, 100μm. **(B)** The ratio of CD16^+^ cells to whole Iba^+^ cells. All data were presented as the means ± SEM. n=4. ## *p*<0.01, and *** *p*<0.001.


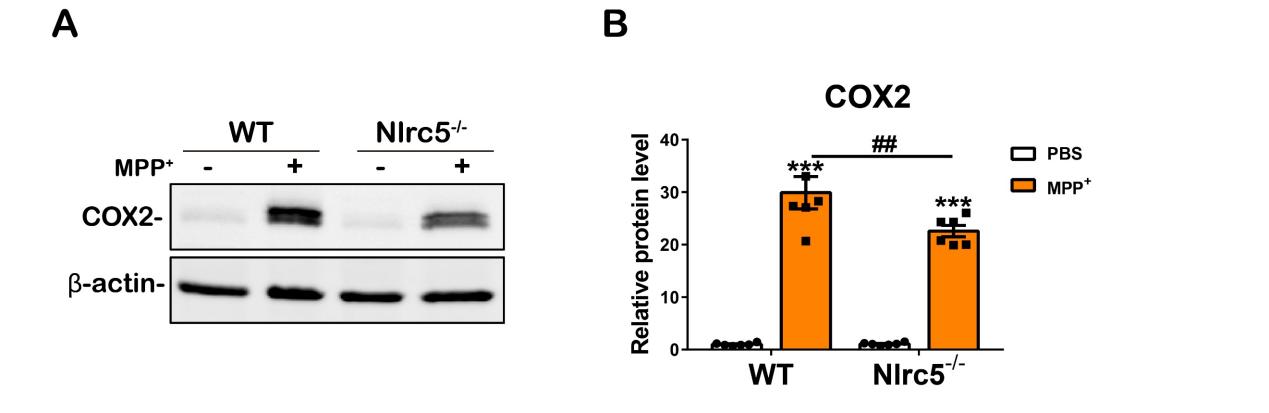


**Figure S4. The expression of COX2 proteins in mixed glial cells at 24h after MPP^+^ (1 mM) treatment. (A)** Immunoblotting of COX2 proteins. **(B)** Statistical analysis of the COX2 protein levels. All data were presented as the means ± SEM. n=4-6. **##** *p*<0.01, ****p*<0.001.


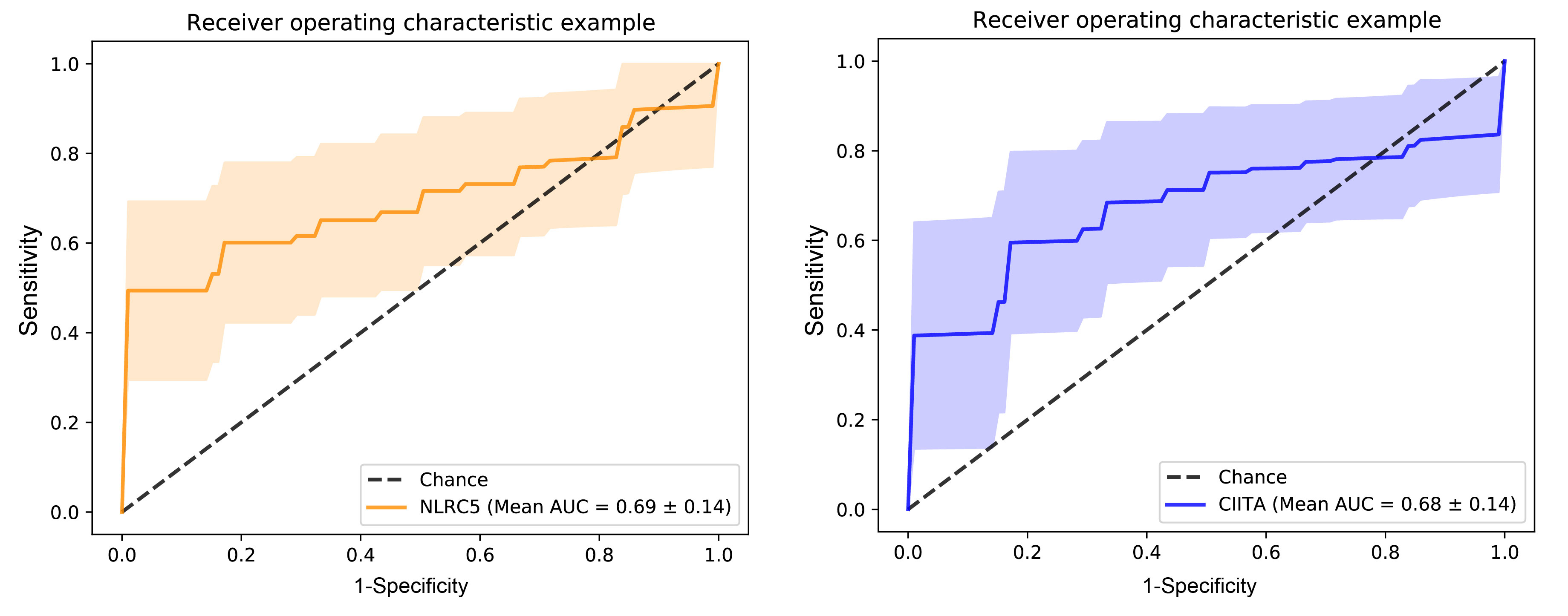


**Figure S5. Receiver operating characteristic example of NLRC5 and CIITA in PD patient blood.**


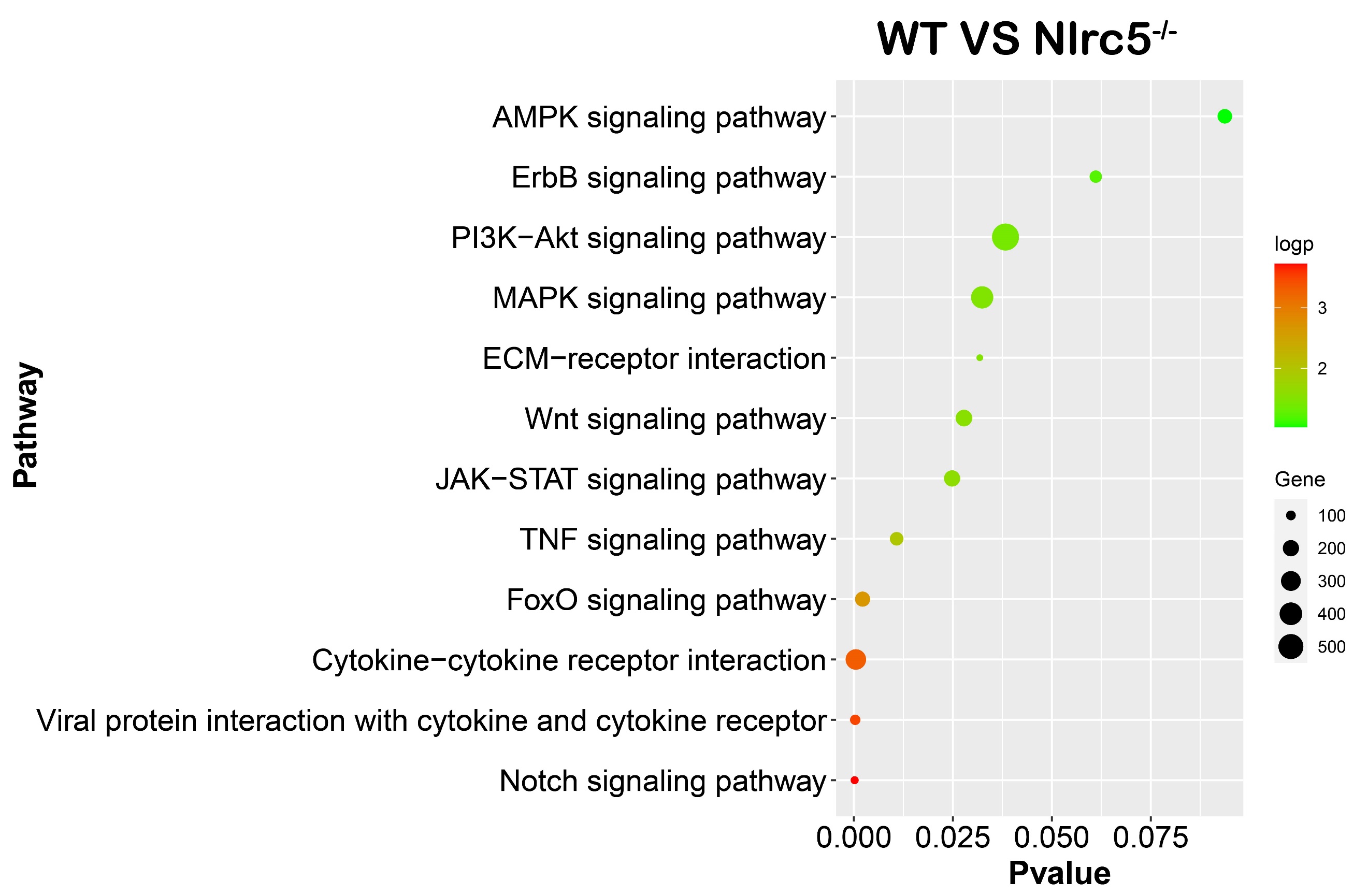


**Figure S6. Enriched KEGG pathway analysis of transcripts in the striatum.** The size of spots corresponds to the numbers of genes, and the color of spots represents *p* value.


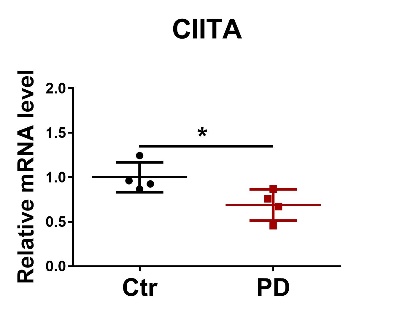


**Figure S7.** **Analysis of *CIITA* expression profile in the GEO database (GDS5646 ILMN_1773363).** Analysis of peripheral blood of untreated patients with stage 1 Parkinson's disease. Data were presented as the means ± SEM. Statistical analyses were performed with Student’s t test. n=4. ***** *p*<0.05.


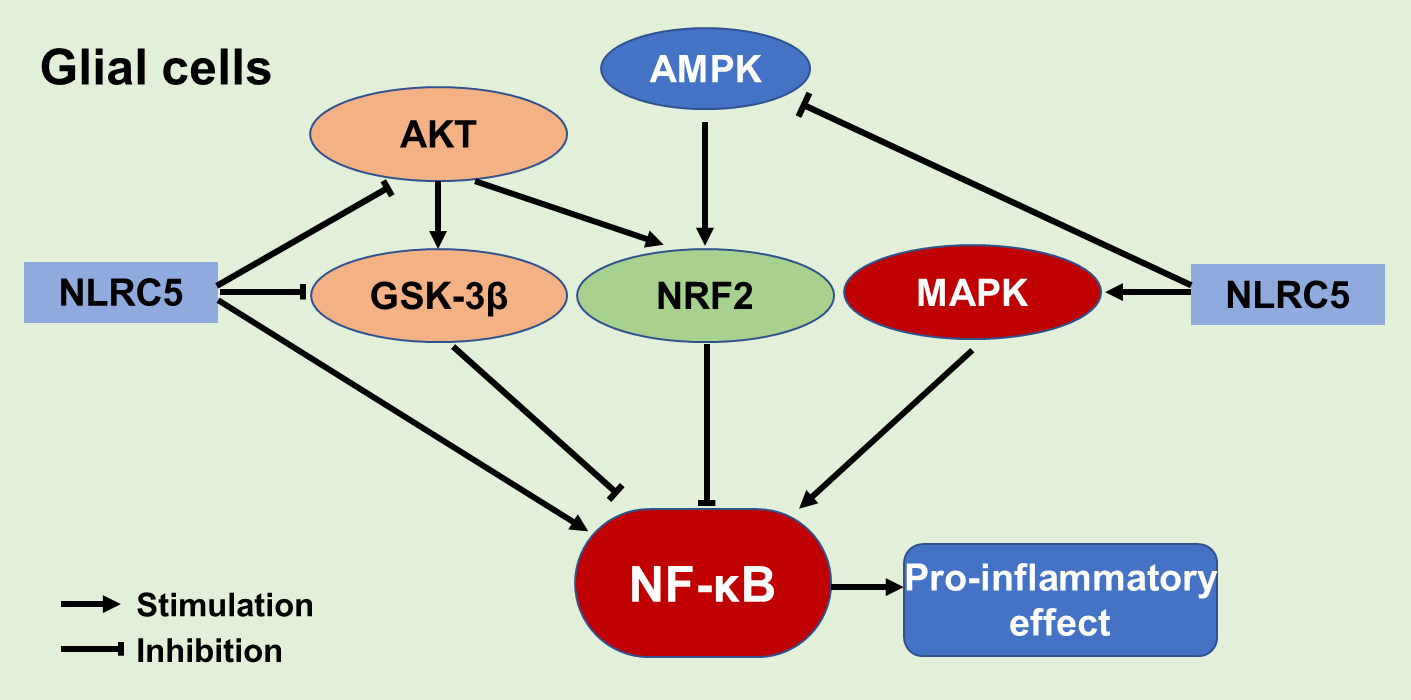

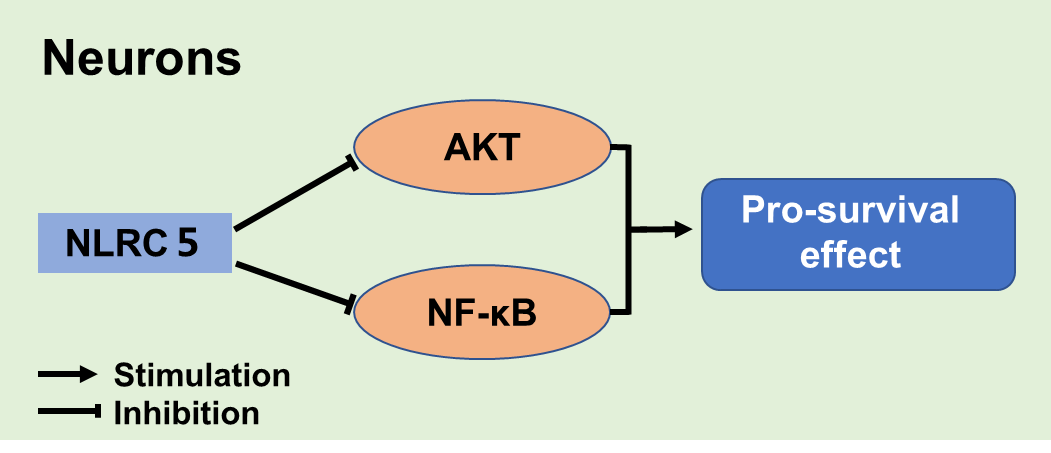


**Figure S8. The signal pathways regulating pro-inflammatory response in glial cells and survival in neurons.**

**Supplementary tables**

**Table S1. Primers used in the RT-qPCR.**

**Mouse genes**

| Primers | Sequence (5’ - 3’) |
| --- | --- |
| ASC F | TGCTTAGAGACATGGGCTTAC |
| ASC R | CAATGAGTGCTTGCCTGTG |
| BAX F | TGCAGAGGATGATTGCTGAC |
| BAX R | GAGGACTCCAGCCACAAAGA |
| Bcl-2 F | ACTGGAGAGTGCTGAAGATTG |
| Bcl-2 R | CATCCCTGAAGAGTTCCTCCAC |
| BDNF F | TCATACTTCGGTTGCATGAAGG |
| BDNF R | AGACCTCTCGAACCTGCCC |
| C1q F | AGCATCCAGTTTGATCGGAC |
| C1q R | CTTCAGCCACTGTCCATACTAG |
| C3 F | GGGCTGTTAAATGGTTGATTCTG |
| C3 R | GATGAGGACGAAGGCTGTG |
| CD40 F | GTGCAGTGACAAACAGTACCTC |
| CD40 R | GAAGCCCTTGATTGGGTTCACAGT |
| CIITA F | TTCTGGACTGGGAAACCTCGTG |
| CIITA R | ATCCAGGTCTTCCACATCCTTT |
| COX2 F | GTTCATCCCTGACCCCCAAG |
| COX2 R | ACTCTGTTGTGCTCCCGAAG |
| Drd2 F | GCAATGTGCTGGTGTGCATGGC |
| Drd2 R | GAGCAGTGGGCAAGAGATGGTG |
| IL-10 F | AGCCGGGAAGACAATAACTG |
| IL-10 R | GGAGTCGGTTAGCAGTATGTTG |
| IL-18 F | CTTCGTTGACAAAAGACAGCC |
| IL-18 R | CACAGCCAGTCCTCTTACTTC |
| IL-1α F | TTCTGCCATTGACCATCTCTC |
| IL-1α R | GTTGCTTGACGTTGCTGATAC |
| IL-1β F | GCAACTGTTCCTGAACTC |
| IL-1β R | CTCGGAGCCTGTAGTGCA |
| IL-4 F | GCATTTTGAACGAGGTCACAG |
| IL-4 R | TGGAAGCCCTACAGACGAG |
| IL-6 F | CCTACCCCAATTTCCAATGCT |
| IL-6 R | TATTTTCTGACCACAGTGAGGAAT |
| iNOS F | CCCTTCCGAAGTTTCTGGCAGCAGC |
| iNOS R | GGCTGTCAGAGCCTCGTGGCTTTGG |
| MHCI F | GTCCTTCAGCAAGGACTGGTC |
| MHCI R | TGGATTTGTAATTAAGCAGGTTC |
| MHCII F | ACACGGTGTGCAGACACAA |
| MHCII R | TCAGGCTGGGATGCTCC |
| NLRC5 F | AGAGCTATGGGTCCTCACCCCGTC |
| NLRC5 R | CTGAGCATCCAAGGGTGCTGTAG |
| NLRP3 F | ATGGGTTTGCTGGGATATCTC |
| NLRP3 R | GCGTTCCTGTCCTTGATAGAG |
| NOX1 F | AGGTCGTGATTACCAAGGTTGTC |
| NOX1 R | AAGCCTCGCTTCCTCATCTG |
| NOX2 F | AGCTATGAGGTGGTGATGTTAGTGG |
| NOX2 R | CACAATATTTGTACCAGACAGACTTGAG |
| TGF-β F | CCTGAGTGGCTGTCTTTTGA |
| TGF-β R | CGTGGAGTTTGTTATCTTTGCTG |
| TNF-α F | CACGCTCTTCTGTCTACTGAACTTC |
| TNF-α R | GCAGCCTTGTCCCTTGAAGAGAACC |
| β-actin F | CAGGATGCAGAAGGAGATTAC |
| β-actin R | AACGCAGCTCAGTAACAGTC |

**Human genes**

| Primers | Sequence (5’ - 3’) |
| --- | --- |
| IL-1β F | ACCTTCTTTCCCTTCATCTTTG |
| IL-1β R | CTTGTTGCTCCATATCCTGTCC |
| CⅡTA F | CCTGACCTCCCGAGCAAACA |
| CⅡTA R | CCACCTCCACTAGGATGCCA |
| HLA-A F | CAAGGATTACATCGCCCTGAAC |
| HLA-A R | GAGATGGGGTGGTGGGTCATAT |
| HLA-DR F | CAGGCGAGTTTATGTTTGAC |
| HLA-DR R | CCACAGGGCTGTTTGTGAGC |
| NLRC5 F | AGGAAGTAAAATCACTGCCCGAGG |
| NLRC5 R | AGCAGAGTAGGGTTGACACGCA |
| β-actin F | CAGGATGCAGAAGGAGATTAC |
| β-actin R | AACGCAGCTCAGTAACAGTC |

**Table S2. Demographic and clinical profiles of PD patients and control groups.**

**
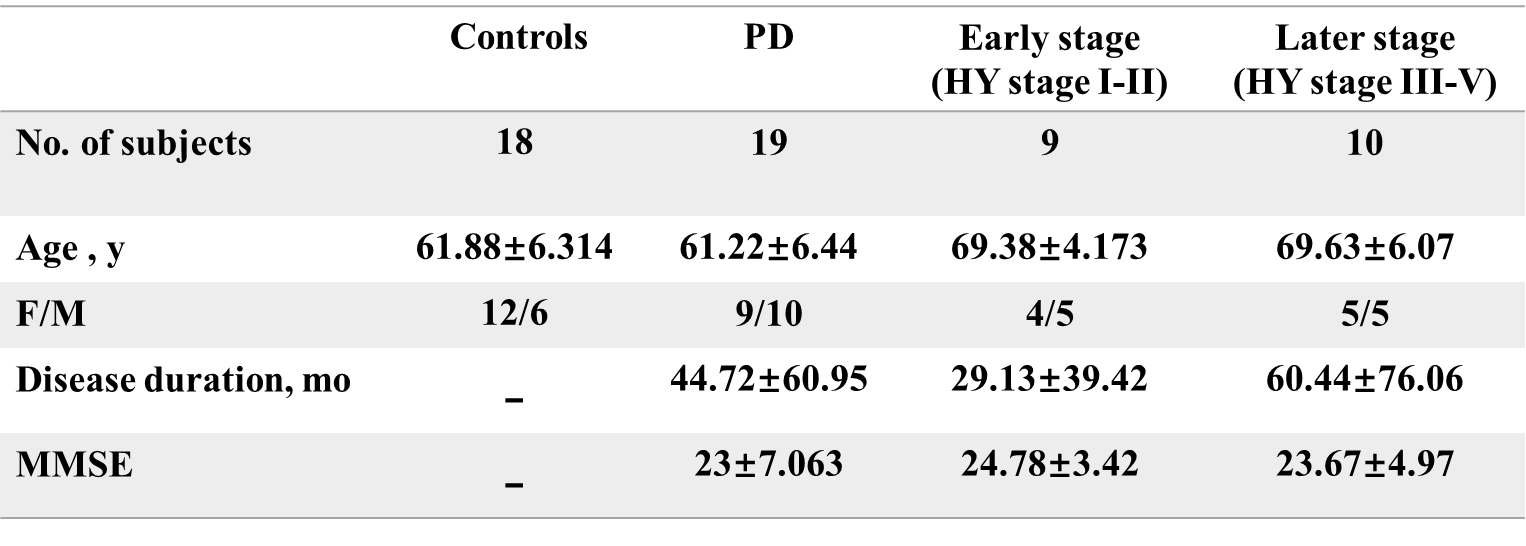
**

Abbreviations: PD =Parkinson’s disease; mo =month; MMSE =Mini Mental State Examination. The data are presented as means ±SD.
